# Training Willpower: Reducing Costs and Valuing Effort

**DOI:** 10.3389/fnins.2022.699817

**Published:** 2022-04-28

**Authors:** Michel Audiffren, Nathalie André, Roy F. Baumeister

**Affiliations:** ^1^Research Centre on Cognition and Learning, Centre National de la Recherche Scientifique, University of Poitiers, Poitiers, France; ^2^School of Psychology, The University of Queensland, St Lucia, QLD, Australia

**Keywords:** cognitive training, effort, executive functions, transfer, exercise training, effortful control, self-control, mindfulness training

## Abstract

The integrative model of effortful control presented in a previous article aimed to specify the neurophysiological bases of mental effort. This model assumes that effort reflects three different inter-related aspects of the same adaptive function. First, a mechanism anchored in the salience network that makes decisions about the effort that should be engaged in the current task in view of costs and benefits associated with the achievement of the task goal. Second, a top-down control signal generated by the mechanism of effort that modulates neuronal activity in brain regions involved in the current task to filter pertinent information. Third, a feeling that emerges in awareness during effortful tasks and reflects the costs associated with goal-directed behavior. The aim of the present article is to complete this model by proposing that the capacity to exert effortful control can be improved through training programs. Two main questions relative to this possible strengthening of willpower are addressed in this paper. The first question concerns the existence of empirical evidence that supports gains in effortful control capacity through training. We conducted a review of 63 meta-analyses that shows training programs are effective in improving performance in effortful tasks tapping executive functions and/or self-control with a small to large effect size. Moreover, physical and mindfulness exercises could be two promising training methods that would deserve to be included in training programs aiming to strengthen willpower. The second question concerns the neural mechanisms that could explain these gains in effortful control capacity. Two plausible brain mechanisms are proposed: (1) a decrease in effort costs combined with a greater efficiency of brain regions involved in the task and (2) an increase in the value of effort through operant conditioning in the context of high effort and high reward. The first mechanism supports the hypothesis of a strengthening of the capacity to exert effortful control whereas the second mechanism supports the hypothesis of an increase in the motivation to exert this control. In the last part of the article, we made several recommendations to improve the effectiveness of interventional studies aiming to train this adaptive function.

“*Keep the faculty of effort alive in you by a little gratuitous exercise every day.*”

James (1918, p. 127)

## Introduction

In daily life, our behavior mainly depends on routinized, automatic and unconscious processes (Ouellette and Wood, 1998; Kahneman, 2011). However, in some cases, effortful control is required to perform a more demanding task, such as maintaining concentration on complex problem solving (e.g., academic tasks), sustaining attention on infrequent cues (e.g., vigilance tasks), repressing immediate impulses to secure delayed benefits or avoid expected costs (e.g., self-control situations), or exercising at an uncomfortable intensity (e.g., sport and rehabilitation situations). Effortful control is deliberate, costly and exerted over brain areas involved in the achievement of a task goal (André et al., 2019; Müller and Apps, 2019). Effortful control is the product of the activity of the mechanism of effort anchored in the salience network (for more details, see André et al., 2019), which includes the dorsal anterior cingulate cortex and the anterior insula (Seeley et al., 2007). The metaphor of the steering wheel (Bargh, 1997; Baumeister and Sommer, 1997) is relevant and illustrates the importance of effortful control in behavior: Even if a car is driven straight-ahead 95% of the time (thus no need for steering), a car without a steering wheel is not 95% as good as a car with one.

People who have a high capacity to exert effortful control are more likely to perform better in work, school and sport situations that require controlled attention or self-control. On the contrary, people who have a low dispositional capacity to exert effortful control, such as individuals with addictions, obsessive-compulsive disorder or attention-deficit hyperactivity disorder, generally present difficulties to regulate intrusive thoughts and emotions and to delay rewards (Pinto et al., 2014; Lugo-Candelas et al., 2017; Eichholz et al., 2020; Lim et al., 2020). In fact, developmental studies have shown that effortful control capacity in childhood predicts academic achievement, physical health, substance dependence, personal finances, antisocial behaviors and criminal offending outcomes later in life (Tarter et al., 2003; Moffitt et al., 2011; Liew, 2012; Fergusson et al., 2013; Daly et al., 2015; Holmes, 2018; for a review see Robson et al., 2020). Strengthening the capacity to exert effortful control through training could be a good way to improve quality of life and well-being of individuals, particularly those who have a low capacity. The term ‘capacity’ refers here to the ability or skill to exert effortful control rather than the maximum amount of resources devoted to effortful control. The aim of the present article is to show that the capacity to exert effortful control is trainable and to propose two plausible neurophysiological mechanisms supporting these durable changes in capacity.

The strength model of self-control (Baumeister et al., 2007, 2018) proposes that self-control could be strengthened through training. Taking the metaphor of the muscle, this model assumes that regular exertions of self-control can improve willpower strength and stamina, just as exercise training can strengthen muscles. The mechanisms underlying these gains in self-control would be an improvement in the self-regulatory general core capacity, i.e., increasing available self-control resources (Oaten and Cheng, 2006). Another important prediction of this model is that the improvements in the general capacity induced by the training program can be extended to other spheres of self-regulation unrelated to what had been practiced (Baumeister et al., 2006). In support of this model, two recent meta-analyses showed that self-control training is effective at strengthening the ability to self-regulate (Friese et al., 2017; Beames et al., 2018).

The strength model of self-control makes a last important prediction: the capacity to exert effortful control can be temporarily weakened after the performance of a first effortful task. This phenomenon called ‘ego depletion effect’ was recently challenged regarding its actual existence (Carter et al., 2015; Vadillo, 2019), and replicability (Xu et al., 2014; Hagger et al., 2016; Lurquin et al., 2016; Osgood, 2017; Alós-Ferrer et al., 2019; Vohs et al., 2021). This debate, which certain researchers considered as closed, is beyond the scope of this paper. But, what does it mean for the present theory if the ego depletion effect is so small that it is practically impossible to study? Strengthening willpower through training should increase the ability to compensate for a temporary weakening of the capacity to exert effortful control (i.e., an ego depletion effect). Consequently, any reader could think that it would be useless to study the possible strengthening of willpower through training if the ego depletion effect does not exist or is negligible.

Three arguments justify the pertinence of studying the improvement of the capacity to exert effortful control through training in spite of this questioning about ego depletion. First, denying a possible transient weakening of the capacity to exert effortful control after a long and intense use of this capacity is ignoring all the literature on cognitive, mental and central fatigue. Cognitive fatigue is generally evidenced in vigilance tasks by a decrement of performance with time-on-task (Mackworth, 1964; Boksem et al., 2005; Ackerman, 2011). In other respect, sport sciences are interested in the impact of mental or central fatigue induced by long and highly demanding cognitive tasks on sport performance. Two systematic reviews conducted on this topic showed a consistent effect of cognitive fatigue on endurance performance (Van Cutsem et al., 2017; Pageaux and Lepers, 2018). Moreover, a recent study showed that performance decreased with time-on-task during a classical depleting task; i.e., the ‘e’ letter task (Arber et al., 2017).

Second, willpower is the capacity to exert effortful control in spite of high costs (Baumeister and Tierney, 2011). As we will see further, different categories of costs are involved in effort-based decision making (i.e., decision about the amount of effortful control dedicated to the achievement of the task goal). Ego depletion and cognitive fatigue belong to the same category of costs: a temporary weakening of the capacity to exert effortful control that requires a compensatory investment in effortful control to maintain performance (André et al., 2019). Other categories of costs can modulate the effort-based decision making, such as the pain associated with the achievement of the task goal (e.g., muscle pain while carrying out a resistance exercise) or the risk of repeated failures (e.g., ego threat or threat to the physical integrity associated with task failures). In this perspective, strengthening willpower allows to cope with a large variety of stressful situations, including fatiguing tasks, painful tasks and risky tasks. For instance, a long-distance runner (e.g., ultra-marathon) has to cope with cognitive fatigue, muscular fatigue, and muscular pain; i.e., the athlete has to maintain an effortful control in spite of these costs if he/she wants to succeed. Consequently, even if the cognitive fatigue associated to the task is negligible, a successful coping with the other constraints of the task justify to train willpower.

Third, the transient weakening and the durable strengthening of the capacity to exert effortful control rely on two distinct neurobiological mechanisms that can be studied separately. As suggested by several authors, the temporary weakening of the capacity to exert effortful control relies on a short-term synaptic mechanism induced by an accumulation of adenosine in prefrontal brain regions involved in the ongoing task (Martin et al., 2018; André et al., 2019). By contrast, as we will see further, the durable strengthening of the capacity to exert effortful control relies on long-term synaptic mechanisms modifying the efficacy of glutamatergic synapses involved in the circuitry connecting the anterior cingulate cortex with brain structures computing costs and benefits (see the section “Neural Bases of Gains in Effortful Control Capacity through Training”). These two phenomena relying on two distinct neurobiological mechanisms, the existence or non-existence of the former does not in any way affect the existence or non-existence of the later, and reciprocally.

The concept of ‘resources’ applied to self-control and ego depletion has also been criticized and some authors, such as Michael Inzlicht, preferred to develop a non-resource-based account of the short- and long-term dynamic characteristics of self-control (Inzlicht et al., 2014b). Evidence for this model has not been forthcoming, and indeed the central prediction — that ego depletion manipulations reduce motivation to exert self-control on the dependent measure — has failed repeatedly (see Baumeister and Vohs, 2016).

Concerning the trainability of the capacity to exert effortful control, the alternative theory proposed by Inzlicht et al. (2014a,b) emphasizes that the motivation to exert effortful control can be increased using motivational techniques, such as implementation intentions and motivational interviewing (for a review, Berkman, 2016). In contrast to this model, we make a clear distinction between the capacity to exert effortful control and the motivation to exert effortful control. People can have the ability without being motivated (and vice versa). For example, some studies clearly showed that individuals are sometimes able to engage in effortful control (i.e., a capacity) but decide not to engage (i.e., a motivation) (e.g., Treadway et al., 2009). Therefore, the decision is not made toward the desired rewards but in order to escape the cost of the effort. As mentioned above, capacity refers to the ability to mobilize brain resources dedicated to effortful control, whereas motivation refers to the motive to mobilize these resources. Generally, training programs aim to develop capacities, and motivational techniques help researchers and practitioners increase the motivation and volition of individuals to engage in these effortful interventions and training programs. Michie et al. (2013) identified up to 93 theory-based behavior change techniques (BCTs) aiming to improve adherence to interventions. The use of these techniques is a prerequisite for the success of an intervention, but they are not the heart of the intervention and do not fully explain the improvement in trained capacity. Generally, the tasks and exercises repeatedly practiced by the participants constitute the true active element leading to a change in the capacity to be improved.

The integrative model of effortful control published by the authors in 2019 (André et al., 2019) proposed a theoretical framework based on recent findings in the field of neuroscience that define clearly what is effort and effortful control and which neuronal network underpins the capacity to exert effortful control. It particularly invokes the following contributions from neuroscience: the theory of attentional effort regarding the role of the cholinergic pathway in the generation and maintenance of the effort signal (Sarter et al., 2006), the theory of the dissociation between the salience network and the executive control network (Seeley et al., 2007; Seeley, 2019), the theory of the dynamic network connectivity regarding the short-term neuroplastic mechanisms that can explain a reduction in prefrontal activity following an exposure to stress or fatigue (Arnsten, 2009; Arnsten et al., 2010, 2012), and the theory of the expected value of control concerning the role of the anterior cingulate cortex in effort-based decision making (Shenhav et al., 2013, 2017).

The main proposal of this model is that effort designates three functional parts: (1) a mechanism anchored in the salience network (i.e., the mechanism of effort), which specializes in perceiving and responding to homeostatic and allostatic demands (Seeley, 2019), (2) a control signal (i.e., the effort signal) that is the main product of the mechanism of effort, that oscillates in the theta band (Onton et al., 2005; Sauseng et al., 2007; Kao et al., 2013), and whose the function is to filter information in the brain regions receiving this control signal, (3) a perception that emerges in awareness during effortful tasks (i.e., the perception of effort), which is a secondary product of the mechanism of effort and reflects the costs associated with the goal-directed behavior. Exerting effortful control, i.e., generating the control signal, is the main function of the mechanism of effort.

The strength model of self-control and the integrative model of effortful control share two important predictions: (1) the capacity to exert effortful control can be temporarily weakened when it is overloaded and used during a long period; (2) the capacity to exert effortful control can be durably improved through extensive and adapted training. However, our integrative model of effortful control differs from the strength model of self-control in three important points: (1) the mechanism underpinning the transient decrease in effortful control capacity (i.e., ego depletion effect or cognitive fatigue effect) is not viewed as the depletion of a resource, but as the weakening of the capacity of a neural system to generate a control signal because of a short-term synaptic mechanism induced by an accumulation of adenosine in prefrontal brain regions involved in the ongoing task, (2) predictions are made at the behavioral and neurophysiological levels and not only at the behavioral level (e.g., durable increase of performance accompanied by a durable increase in between-network connectivity with training), (3) the general core capacity that can be temporarily weakened through intensive use and durably strengthened through training is anchored in the salience network and not in the executive control network that underpins inhibitory control.

The present article focuses on the mechanisms leading to improvements in the capacity to exert effortful control. Motivational techniques are viewed here as moderators that facilitate the engagement of effortful control in training tasks throughout the entire duration of the intervention. The modulatory influence of these moderators on mechanisms leading to an increase in the engagement of effortful control is beyond the scope of this paper.

More specifically, the present paper aims to describe the hypothetical neurophysiological mechanisms that could underpin improvements in the capacity to exert effortful control. Arguing that training increases the amount of available resources (i.e., the capacity of a tank) is not a sufficient level of explanation to improve the methodology, efficacy, and effectiveness of effortful control interventions. This paper tries to answer the two following questions: Is there clear evidence for improvements in effortful control capacity with training? And if so, which durable changes in brain functioning explain these increments in effort capacity?

The following sections provide answers to these questions. In the first section “Definitions,” we present several interrelated concepts that are the object of the training. In the second section “Improvements in Effortful Control with Practice: An Umbrella Review of Meta-Analytic Reviews,” we summarize the main results of several meta-analytic reviews examining the effects of training on the capacity to exert effortful control. We discuss the significance and the size of this effect as a function of several moderators, such as the duration of the intervention and the type of exercises used to train the capacity to exert effortful control. We also address the issue concerning the generalizability/transferability of gains in effortful control capacity. Then, in the third section “Neural Bases of Gains in Effortful Control Capacity through Training,” we describe two brain mechanisms that could explain these training effects. Finally, in the last section “Challenging the Trainability of Effortful Control Capacity,” we formulate a series of recommendations to examine these training effects in the future.

## Definitions

As mentioned in the previous section, a gain in capacity in effortful control can be very beneficial for an individual to increase his/her likelihood of success in personal achievement. In this section, we present the main concepts that constitute the target of the training interventions.

Two broad categories of training programs that are able to improve effortful control capacity have been identified (Beames et al., 2018). The first category of training programs aims to improve executive functions, whereas the second category aims to strengthen self-control, willpower or self-regulation. The following paragraphs will help the reader to disentangle the links between all these closely related concepts and then to understand more clearly how they fit together.

The concept of executive functions (EFs) comes from cognitive psychology and designates high-level cognitive functions anchored in the executive control network, which is a frontoparietal network bilaterally involving the dorsolateral prefrontal cortex (DLPFC) and the posterior parietal cortex (Seeley et al., 2007). Executive control must be distinguished from effortful control that is exerted by another large-scale network: the salience network (Seeley et al., 2007; Seeley, 2019). These two networks are both activated as soon as someone is engaged in a cognitive or physical task (i.e., they are task-positive networks) but ensure different functions. The level of activation of these two networks depends, among other things, on the cognitive load of the ongoing task (Paus et al., 1998). Executive control allows individuals to mentally shift through ideas, to reason before acting, to cope with novel and unexpected challenges, to resist temptations and to stay focused on a specific goal (Diamond, 2013), whereas effortful control helps targeted brain regions involved in the ongoing task to keep the focus on relevant task features (André et al., 2019). The salience network and the executive control network are bidirectionally interconnected. Effortful control enhances executive functioning whereas executive functions send cost signals to the salience network that generates effortful control according to a cost/benefit decision-making.

Miyake et al. (2000) identified three main separable EFs that share commonalities: (a) shifting between tasks or mental sets, (b) updating and monitoring of working memory representations, and (c) inhibition of dominant or prepotent responses. The first component of EFs is also called ‘cognitive flexibility’ and corresponds to the ability to shift from one mental set to another mental set, from one set of guidelines for action to a different set (e.g., shifting from a status of an offensive player to a status of defender in basket-ball as soon as the ball is caught by the opponents). The second component is the ability to maintain, refresh and manipulate relevant information in working memory (e.g., performing the mental rotation of the representation of an object). The third component, also called ‘inhibitory control,’ ‘intentional inhibition,’ or ‘controlled inhibition,’ is the ability to repress or stop prepotent impulses, unwanted and intrusive thoughts, embarrassing emotions, or automatic motor responses.

More recently, Zelazo and Carlson (2012) introduced a new taxonomy of EFs, taking into account the context in which participants exert executive control. These authors proposed distinguishing cool EFs solicited and assessed in emotionally neutral contexts, such as laboratory settings, and hot EFs involved in motivationally and emotionally significant high-stakes situations, such as multiplayer online role-playing games or real social situations in daily life. As discussed later, these two categories of EFs are used in different types of interventions aiming to develop effortful control capacity.

Inhibitory control presents many similarities with the concept of self-control used in social psychology when the latter is more restrictively designated as the ability to follow rules or inhibit immediate desires so as to delay gratification (e.g., Muraven and Baumeister, 2000, p. 247), as well as to interrupt undesired behavioral tendencies and refrain from acting on them (e.g., Tangney et al., 2004, p. 274). However, the concept of self-control has a larger meaning when it is used interchangeably with the concept of self-regulation (Baumeister and Vohs, 2016, p. 70). Based on this larger meaning, it refers to the ability to voluntarily regulate attention, emotion, and behavior in the service of more highly valued goals and represents the deliberate, conscious, effortful subset of self-regulation (Baumeister et al., 2007, p. 351).

Willpower is a folk term referring to mental energy that is expended in difficult acts of self-control, such as resisting temptation and delaying gratification (Baumeister and Tierney, 2011). It is often used in connection with making current sacrifices for the sake of long-term benefits and goals. In the same way, effortful control invokes executive functions and helps to inhibit behavioral impulses so as to regulate emotions and behaviors, thereby enabling people to adjust to situations in flexible, adaptive fashion (André et al., 2019). The common theme is that the Self exerts effort to regulate its own responses to produce preferred outcomes. Philosophers have identified a set of virtues or skills associated with a strong willpower, such as persistence, endurance, perseverance, resoluteness and patience (Roberts, 1984; Steutel, 1999; Szutta, 2020). All these virtues help an individual to remain focused on his/her intended goals and to facilitate their achievement. In the framework of the integrative model of effortful control, we assume that willpower is the capacity to exert effortful control in difficult situations, such as sustaining attention in boring vigilance task or maintaining a high intensity of exercise in spite of fatigue and pain.

One important commonality between EFs and self-control, in both its more restrictive and larger meaning, is that all these high-level cognitive functions require effortful control. Based on the framework of the integrative model of effortful control (André et al., 2019), we assume here, that the self-regulatory general core capacity, which can be temporarily weakened through intensive use and durably strengthened through training, corresponds to the effortful control capacity ensured by the salience network. In addition, we assume that the good functioning of the executive control network, which underpins EFs, depends directly on the effortful control exerted by the salience network.

Effortful control is not conceived here as a depletable resource but as a control signal that can be weakened and/or deteriorated under the effect of fatigue (for more information about the mechanisms underpinning the possible weakening of this control signal, see André et al., 2019). In the same way, effortful control capacity can be conceived as the function of the mechanism of effort to generate this control signal, which can be directly assessed by measuring spectral power of theta-wave activity above prefrontal areas (e.g., Cavanagh and Franck, 2014; Fairclough and Ewing, 2017). Higher the density of prefrontal theta-wave activity is, higher the engagement in effortful control. Exerting effortful control means that the organism needs to mobilize energy, and the activation of the sympathetic system is closely linked to the exertion of effortful control (Critchley, 2005). In that way, indexes of sympathetic activity, such as pupil size and pre-ejection period, are used as indirect measures of effortful control (Richter et al., 2008; van der Wel and van Steenbergen, 2018).

Based on the above, we can make a series of hypotheses: (1) the capacity to maintain a high level of effortful control over time in spite of fatigue or pain (i.e., a high level of concentration or effort engagement) can be strengthened through training programs involving effortful activities; (2) training programs targeting self-control or EFs stimulate effortful control and can strengthen this general capacity; (3) training programs more specifically targeting EFs lead to several synergistic effects: a strengthening of the effortful control capacity through durable changes within the salience network, a strengthening of the EFs through durable changes within the executive control network and a strengthening of the connectivity between these two networks.

Finally, the notion of transfer is central in the cognitive training literature and related to the generalizability of the gain obtained through extensive practice. Transfer distance refers to the similarities between the trained tasks and the tasks used to demonstrate a gain in performance at the end of the intervention (i.e., the principal outcome). Two types of transfer can be distinguished: (a) ‘near-transfer’ effects when trained tasks and postintervention untrained tasks are similar, (b) ‘far-transfer’ effects when trained tasks and postintervention tasks are dissimilar. The ultimate goal of interventions targeting effortful control capacity is to promote far-transfer effects because the gain in this general capacity should ideally be transferable to a broad range of everyday functional activities.

## Improvements in Effortful Control With Practice: An Umbrella Review of Meta-Analytic Reviews

In this section, we summarize the main results of meta-analyses focusing on the long-term effects of different types of training methods stimulating effortful control. As mentioned earlier, Beames et al. (2018) distinguished two main categories of training methods: methods focusing on improving executive functions and methods focusing on strengthening self-control. Each following subsection addresses three important issues: the effectiveness of the training method to increase performance in effortful tasks that engage EFs or self-control, the stability of these gains once training stops and the generalizability/transferability of these gains. The method used to select, extract information and evaluate for risk of bias in these meta-analyses is detailed in the Supplementary Material.

### Interventions Targeting Executive Functions

A very large number of studies have examined the effectiveness of miscellaneous training methods on EFs. Four main categories of training methods can be distinguished: process-based cognitive training, physical training, video-game training, and mindfulness training. Process-based cognitive training aims to directly increase the efficiency of specific cognitive processes, such as core EFs, through extensive repeated practice of affectively neutral computerized and/or manual cognitive tasks tapping the targeted cognitive process. Physical training aims to improve higher cognitive functions, such as EFs and episodic memory, through the regular practice of aerobic, resistance and/or coordinative exercises. Video game training stimulates miscellaneous cognitive functions, such as hot EFs, through video games, exergames or serious games that generally involve motivationally salient contexts or simulated social contexts generating heightened emotion. Mindfulness training is the regular practice of exercises maintaining attention to the current situation while concurrently acknowledging any thoughts or feelings that arise in consciousness (Bishop et al., 2004).

### Process-Based Cognitive Training Interventions

Table 1 summarizes the results of sixteen meta-analyses published from 2011 to 2021, which focused on the effect of process-based cognitive training on EFs (near-transfer effects) and other far-transfer outcomes. Strategy-based training methods were not taken into consideration because they focus more heavily on compensatory rather than restorative methods, bypassing deficient cognitive processes and teaching alternative approaches to achieving goals (Mowszowski et al., 2016). For instance, strategy-based training programs aiming to compensate for memory deficits typically include internal techniques (e.g., categorizing or visualizing information to be remembered, encoding through multiple sensory channels) and external techniques (e.g., using environmental cues, calendars or memory notebooks).

**TABLE 1:** Meta-analyses reporting effect sizes of process-based cognitive training on executive functions and other far-transfer outcomes.

References	Trained functions	NO studies (A/B)	Population	Duration of interventions	Results
Karch et al., 2013	Attention, executive functions, long-term memory, visuospatial abilities, working memory	NT: 11/22 FT: 4/22	Children and adolescents (4–20 years)	4–15 weeks *M* = 8.7 weeks	NT: *d* = 0.17 ns FT: *d* = 0.29 ns
Rapport et al., 2013 ^†^	Attention, executive functions	NT: 3/17 FT: 9/17	Children and adolescents with ADHD	2–16 weeks *M* = 7.3 weeks	NT: *d* = 0.06 ns FT: *d* = 0.28*
Lampit et al., 2014	Attention, multidomain, processing speed, video game, working memory	29/51	Healthy older adults (≥60 years)	2.5–16 weeks *M* = 7.4 weeks	*g* = 0.09 ns
Cortese et al., 2015	Attention, executive functions, memory, multidomain, working memory	EFR: 6/16 WMvi: 5/16 WMve: 8/16 IC: 6/16	Children and adolescents with ADHD (3–18 years)	4–20 weeks *M* = 7.5 weeks	EFR: SMD = 0.35* WMvi: SMD = 0.47* WMve: SMD = 0.52* IC: SMD = 0.07 ns
Lawrence et al., 2017	Attention, executive functions, memory, psychomotor speed, visuospatial abilities, working memory	8/11	Older adults with Parkinson’s disease	1–7 weeks *M* = 4.7 weeks	*g* = 0.42*
Sherman et al., 2017 ^†^	Memory, multidomain, processing speed, strategy-based training, working memory	13/26	Older adults with MCI (mean age = 72.6 years)	2–24 weeks *M* = 12.1 weeks	*g* = 0.575*
Soveri et al., 2017	Updating of working memory	33/33	Young, middle-aged and older adults (18–84 years)	1–15 h *M* = 6.4 h	N-back: *g* = 0.62* WM: *g* = 0.24* CC: *g* = 0.16* Gf: *g* = 0.16*
Webb et al., 2018	Attention, multidomain, processing speed, video game, working memory	EF: 29/51 UWM: 7/51 CF: 22/51 IC: 19/51	Healthy older adults (≥60 years)	2–16 weeks *M* = 7.5 weeks	EF: *g* = 0.17* UWM: *g* = 0.005 ns CF: *g* = 0.17* IC: *g* = 0.16*
Lampit et al., 2019 ^†^	Attention, executive functions, processing speed, memory	14/20	Middle-aged adults with multiple sclerosis (mean age = 46.9 years)	4–12 weeks *M* = 8.2 weeks	*g* = 0.29*
Nguyen et al., 2019 ^†^	Executive functions, working memory	TO: 24/64 NT: 55/64 FT: 57/64	Healthy older adults (53–95 years)	1–27 weeks *M* = 7.0 weeks	TO: *g* = 1.00* NT: *g* = 0.26* FT: *g* = 0.22*
Takacs and Kassai, 2019 ^†^	Attention, executive functions, long-term memory, reasoning, working memory	WM: 34/90 IC: 31/90 CF: 20/90	Children (≤12 years)	1–12 weeks *M* = 5.4 weeks	WM: *g* = 0.451* IC: *g* = 0.213* CF: *g* = 0.31*
Zhang et al., 2019	Attention, long-term memory, multidomain, processing speed, working memory	11/18	Older adults with MCI (mean age = 73.4 years)	2–26 weeks *M* = 10.5 weeks	*g* = 0.20 ns
Basak et al., 2020	Executive functions, memory, multidomain, processing speed, reasoning	MCI: 33/54 HA: 116/161 NT: 41/215 FT: 38/215 AO: 8/215	Older adults with or without MCI (≥60 years)	0.5–270 h *M* = 23.3 h 1–90 weeks *M* = 8.3 weeks	MCI: *g* = 0.29* HA: *g* = 0.27* NT: *g* = 0.44* FT: *g* = 0.31* AO: *g* = 0.18 ns
Pauli Pott et al., 2020	Inhibitory control, cognitive flexibility, working memory	WM: 23/35 cool IC: 26/35 hot IC: 4/35 CF: 12/35	ADHD children (mean age = 5.0 years)	1–52 weeks *M* = 11.3 weeks	WM: *d* = 0.46* cool IC: *d* = 0.30* hot IC: *d* = 0.33* CF: *d* = 0.47*
Scionti et al., 2020	Executive functions, reasoning, working memory	NT: 30/32 FT: 16/32 AO: 13/32	Children (3–6 years)	2.5–54.8 h *M* = 11.4 h	NT: *g* = 0.352* FT: *g* = 0.318* AO: *g* = 0.10 ns
Nguyen et al., 2021	Commercial multidomain cognitive training programs	25/43	Healthy older adults (mean age = 70.6 years)	6.7–80 h *M* = 18.3 h 2–16 weeks *M* = 7.4 weeks	*g* = 0.19*

*Significant effect. ^†^The meta-analysis calculated effect sizes for follow-up data. The third column expresses the ratio A/B. The denominator B designates the total number of studies included in the meta-analysis whereas the numerator A designates the number of intervention studies including at least one measurement of executive functions that was used to compute the effect size concerning executive functions. The range and average of intervention durations have been calculated exclusively from studies aiming to train EFs. ADHD, attention-deficit hyperactivity disorder; AO, additional outcomes; CC, cognitive control; CF, cognitive flexibility; CT vs. AC, cognitive training versus active control; CT vs. NI, cognitive training vs. no intervention; EA, executive attention; EF, executive function; EFR, executive function rating; FT, far-transfer effect; Gf, fluid intelligence; HA, healthy aging; IC, inhibitory control; MCI, mild cognitive impairment; NT, near-transfer effect; SMD, standardized mean difference; TO, trained outcomes; UWM, updating of working memory; WM, working memory; WMve, verbal working memory; WMvi, visual working memory; ns, non-significant effect.

The methods used to calculate the effect sizes varied greatly across meta-analyses. The most commonly used methods were Cohen’s *d* (Cohen, 1988) and Hedge’s *g* (Hedges, 1981), but alternative methods to calculate standardized mean difference (SMD) have also been used (e.g., Morris, 2008).

The 16 meta-analyses included in Table 1 principally targeted three populations: children, adolescents and older adults. Ten out of 16 meta-analyses showed a significant and small to moderate effect of process-based cognitive training on near-transfer outcomes (i.e., performance in tasks different from trained tasks but tapping the same cool EFs). By contrast, only four meta-analyses reported a significant effect of process-based cognitive training on far-transfer outcomes (Rapport et al., 2013; Nguyen et al., 2019; Basak et al., 2020; Scionti et al., 2020). However, several categories of far-transfer outcomes must be distinguished. Performance in tasks tapping untrained EFs belongs to the first category of far-transfer outcomes, for instance, the effect of a working-memory training program using n-back tasks on inhibitory control assessed with a Stroop task. Performance in academic or everyday functioning tasks belongs to the second category of far-transfer outcomes (e.g., literacy tasks, calculation tasks). Performance in emotional and social self-regulation tasks (i.e., hot executive functions) belongs to the third category of far-transfer outcomes. Finally, blinded or unblinded subjective ratings of problem behaviors (e.g., inattention, hyperactivity, quick-temperedness and disruptiveness) by a relative, a teacher or a caregiver belong to the fourth category of far-transfer outcomes.

Rapport et al. (2013) showed that programs designed to train working memory, EFs, and attention in children with attention-deficit hyperactivity disorder (ADHD) lead to significant, small magnitude improvements in the first category of outcomes, but non-significant changes for the second and fourth categories of outcomes (i.e., academic achievement measures and blinded behavior ratings, respectively). In the same way, the meta-analysis conducted by Scionti et al. (2020) in preschool children showed that process-based cognitive training programs lead to significant far-transfer benefits in the first category, but not to outcomes belonging to the three other categories. The meta-analysis of Nguyen et al. (2019) focused on far-transfer effects in the first category only and confirmed that these gains can be observed in older adults. Finally, the meta-analysis conducted by Basak et al. (2020) in older adults showed overall significant net gains of process-based cognitive training versus the control conditions on everyday functional outcomes, but these gains were obtained through training programs targeting processing speed.

To sum-up, process-based cognitive training successfully improve EFs with a small to moderate effect size on near-transfer outcomes. However, they generally fail to induce far-transfer outcomes, such as performance in everyday tasks involving EFs or self-control. This last result suggests that process-based cognitive training methods induce gains in cognition that are not sufficiently generalizable and transferable to train willpower.

### Physical Training Interventions

Table 2 summarizes the results of 28 meta-analyses published during the period 2003–2021, which reported the effect sizes of chronic exercise on EFs. These meta-analyses targeted children and adolescents (7 meta-analyses), young and middle-aged adults (7 meta-analyses), and older adults (14 meta-analyses). Seven meta-analyses focused on symptomatic populations (AD, ADHD, chronic brain disorders, and MCI). A large majority of meta-analyses (26 out of 28) showed a significant effect of exercise training on EFs. Among the four meta-analyses with the highest quality score (Karr et al., 2014; Alvarez-Bueno et al., 2017; Biazus-Sehn et al., 2020; Ludyga et al., 2020; *M* = 13.75/16; SD = 0.5), three clearly showed a significant effect of exercise training on EFs. None of these meta-analyses examined the effect of exercise interventions on other secondary effortful control domains.

Two meta-analyses focusing on the effect of interventional studies combining physical and process-based cognitive training on EFs were selected for the present systematic review (Zhu et al., 2016; Guo et al., 2020; see Supplementary Table 3.1). Both of them showed a significant but small effect of these combined interventions on EFs.

### Video Game Training Interventions

Three meta-analyses examining the effect of video game training on EFs (Stanmore et al., 2017; Mura et al., 2018; Mansor et al., 2020) have been selected for the present systematic review. The meta-analysis of Stanmore et al. (2017) reported the results of 17 studies conducted in adults ranging from 17 to 85 years of age. These authors observed a significant effect of exergames on global EFs (*g* = 0.256, 13 studies), cognitive flexibility (*g* = 0.348, 8 studies), and inhibitory control (*g* = 0.90, 5 studies), but a non-significant effect on working memory (4 studies) and problem solving (3 studies). The meta-analysis of Mura et al. (2018) reported the results of 13 intervention studies in persons suffering from neurological disabilities (multiple sclerosis, poststroke hemiparesis, Parkinson’s disease, dementia, dyslexia, and Down syndrome). They showed a significant and positive effect of exergames on EFs (SMD = 0.53, eight studies) but not on attention (seven studies). The meta-analysis conducted by Mansor et al. (2020) included 27 intervention studies and examined the effect of video game training on EFs in older adults. Video game training had no significant effects on attention (8 studies), reasoning (10 studies), cognitive flexibility (15 studies), and inhibitory control (15 studies). By contrast, video game training led to a significant and moderate effect on working memory updating (*g* = 0.37, 19 studies). The duration of video game interventions ranged from 2 to 24 weeks, with an average of 9.4 weeks for the three meta-analyses. The three meta-analyses did not report any other far-transfer outcomes. Supplementary Table 3.2 describes the main characteristics of these three meta-analyses.

### Mindfulness Training Interventions

Finally, eight meta-analyses including randomized controlled studies reporting mean effect sizes of mindfulness training interventions on EFs have been selected in the present systematic review (Chan et al., 2019; Dunning et al., 2019; Cásedas et al., 2020; Poissant et al., 2020; Im et al., 2021; Millett et al., 2021; Verhaeghen, 2021; Yakobi et al., 2021). The characteristics of these meta-analyses are detailed on Supplementary Table 3.3. Two meta-analyses focused on specific populations: the meta-analysis of Chan et al. (2019) on older adults and the meta-analysis of Dunning et al. (2019) on children and adolescents. All the six other meta-analyses mainly concerned young and middle-aged adults. Seven out of these eight meta-analyses showed a significant and small to moderate effect of mindfulness training on EFs (Dunning et al., 2019; Cásedas et al., 2020; Poissant et al., 2020; Im et al., 2021; Millett et al., 2021; Verhaeghen, 2021; Yakobi et al., 2021). The eight meta-analyses shared 31.6% of duplicates. Mindfulness-based programs reported in these meta-analyses were in average shorter than exercise training programs listed in Table 2 (6.6 weeks vs. 23.5 weeks, respectively), but as exercise training programs they provide additional benefits on mental health and well-being (e.g., reduction of anxiety, depression and reactivity to stress).

**TABLE 2:** Meta-analyses reporting an effect of chronic exercise on executive functions.

References	Type of intervention	NO studies (A/B)	NO effects	Duration of interventions	Population	Results
Colcombe and Kramer, 2003	Exercise training	18/18	37	8–144 weeks *M* = 25.3 weeks	Older adults (≥55 years)	*g* = 0.68*
Smith et al., 2010	Exercise training	19/29	19	6–72 weeks *M* = 23.7 weeks	Young and middle-aged adults (≥18 years)	*g* = 0.123*
Hindin and Zelinski, 2012	Extended cognitive training and Aerobic training	17/42	90	8–144 weeks *M* = 29.3 weeks	Older adults (≥55 years)	*d* = 0.459*
Karr et al., 2014	Exercise training	EA: 13/22 PS: 5/22 WM: 8/22 IC: 11/22 VF: 8/22	EA: 20 PS: 6 WM: 14 IC: 17 VF: 11	4–52 weeks *M* = 22.2 weeks	Older adults (≥65 years)	EA: *d* = 0.15* PS: *d* = 0.12 ns WM: *d* = 0.13 ns IC: *d* = 0.06 ns VF: *d* = 0.12 ns
Jackson et al., 2016	Exercise training	8/8	8	8–52 weeks *M* = 27.8 weeks	Children (6–12 years) *M* = 9.4 years	*d* = 0.20*
Alvarez-Bueno et al., 2017	Exercise training	24/36	42	1.5–54 weeks *M* = 22.9 weeks	Children and adolescents (4–18 years)	*d* = 0.20*
Barha et al., 2017	Aerobic training: AT Resistance training: RT Multimodal training: MT	AT: 14/39 RT: 7/39 MT: 11/39	AT: 44 RT: 34 MT: 26	8–96 weeks *M* = 28.8 weeks	Middle-aged adults (≥45 years)	AT: *g* = 2.064* RT: *g* = 0.639* MT: *g* = 0.494*
de Greeff et al., 2018	Exercise training	12/31	15	6–36 weeks *M* = 22.7 weeks	Children (6–12 years)	*g* = 0.24*
Northey et al., 2018	Exercise training	36/39	94	6–52 weeks *M* = 24.5 weeks	Older adults (≥50 years)	SMD = 0.34*
Zhang et al., 2018	Mind-body training	11/19	40	8–40 weeks *M* = 20.2 weeks	Older adults (≥60 years)	0.25 ≤ *g* ≤ 0.65*
Landrigan et al., 2020	Resistance training	16/24	16	4–96 weeks *M* = 28.3 weeks	Young and middle-aged adults (≥18 years)	SMD = 0.39*
Falck et al., 2019	Exercise training	40/47	174	8–104 weeks *M* = 25.1 weeks	Older adults (≥60 years)	*g* = 0.19*
Sanders et al., 2019	Exercise training	22/36	39	4–52 weeks *M* = 24.1 weeks	Young and middle-aged adults with and without MCI (≥18 years)	*d* = 0.25*
Takacs and Kassai, 2019	Exercise training	21/22	22	6–44 weeks *M* = 18.5 weeks	Children (4–12 years)	*g* = 0.16*
Wu et al., 2019	Exercise training	17/32	CF: 13 WM: 10	7–48 weeks *M* = 21.4 weeks	Older adults (*M* = 71.1 years)	CF: MD = 8.80* WM: MD = 0.32*
Xue et al., 2019	Exercise training	18/19	33	5–54 weeks *M* = 24.7 weeks	Children and adolescents (6–17 years)	SMD = 0.20*
Zou et al., 2019	Mind-body training	8/12	9	8–52 weeks *M* = 22.6 weeks	Older adults (≥50 years)	SMD = 0.42*
Biazus-Sehn et al., 2020	Exercise training	15/27	19	6–52 weeks *M* = 24.3 weeks	Older adults with MCI (Mean age = 72.5 years)	SMD = 0.213*
Cai et al., 2020	Taijiquan training	9/19	18	10–52 weeks *M* = 32.6 weeks	Older adults with MCI (Mean age = 71.6 years)	SMD = 0.33*
Chen et al., 2020	Exercise training	33/33	107	4–52 weeks *M* = 25.7 weeks	Older adults (≥50 years)	*g* = 0.21*
Liu et al., 2020	Exercise training	22/22	IC: 15 WM: 14 CF: 8	8–24 weeks *M* = 13.5 weeks	Children and adolescents (5–15 years)	IC: SMD = 0.30* WM: 0.54* CF: SMD = 0.34*
Ludyga et al., 2020	Exercise training	68/80	80	4–52 weeks *M* = 21.4 weeks	Middle-aged and older adults *M* = 47.9 years	*g* = 0.164*
Zhu et al., 2020	Exercise training	12/16	12	12–48 weeks *M* = 20.0 weeks	Older adults with AD (*M* = 76.7 years)	SMD = 0.42*
Dauwan et al., 2021	Exercise training	14/36	14	4–52 weeks *M* = 20.5 weeks	Middle-aged adults with chronic brain disorders (*M* = 55.1 years)	*g* = 0.151*
Huang et al., 2021	Exercise training	26/71	26	6–93 weeks *M* = 26.1 weeks	Older adults with MCI or AD (*M* = 74.3 years)	SMD = 0.39*
Ren et al., 2021	Mind-body training	29/29	29	4–52 weeks *M* = 20.4 weeks	Middle-aged and older adults (*M* = 67.5 years)	SMD = 0.28*
Welsch et al., 2021	Exercise training	9/12	9	8–78 weeks *M* = 17.3 weeks	Children with ADHD (*M* = 9.7 years)	SMD = 0.57 ns
Xiong et al., 2021	Exercise training	25/25	WM: 19 CF: 15 IC: 15	4–56 weeks *M* = 25.4 weeks	Older adults (*M* = 69.9 years)	WM: *g* = 0.127* CF: *g* = 0.511* IC: *g* = 0.136*

*Significant effect. The third column expresses the ratio A/B. The denominator B designates the total number of studies included in the meta-analysis whereas the numerator A designates the number of intervention studies including at least one measurement of executive functions that was used to compute the effect size concerning executive functions. The meta-analysis of Hindin and Zelinski includes 25 extended process-based cognitive training programs and 17 aerobic exercise programs. AD, Alzheimer’s disease; AUS, autism spectrum disorder; EA, executive attention; IC, inhibitory control; MCI, mild cognitive impairment; NO studies, Number of studies included in the calculation of effect size for executive functions/Total number of studies included in the meta-analysis. PS, problem solving; VF, verbal fluency; WM, working memory; SMD, standardized mean difference.

### Interventions Targeting Self-Control

A few interventions have explored the beneficial effects of self-control training on self-control capacity. Self-control interventions do not focus specifically on inhibitory control but generally use a large variety of training tasks involving one or several spheres of self-control described by Hagger et al. (2010), such as volition and social processing. Four meta-analyses examined the effects of self-control training in young adults (Hagger et al., 2010; Inzlicht and Berkman, 2015; Friese et al., 2017; Beames et al., 2018). These meta-analyses have included 33 intervention studies, 11 of which are unpublished. Two other meta-analyses focused on children and adolescents (Piquero et al., 2016; Pandey et al., 2018). Together, they included 41 intervention studies, seven of which were in common and 16% were unpublished studies. All these meta-analyses showed a significant effect of training on self-control capacity. The mean effect size ranged from small (*d* = 0.17, Inzlicht and Berkman, 2015) to large (*d* = 1.07, Hagger et al., 2010) in young adults and was moderate for children and adolescents (*d* = 0.32, Piquero et al., 2016; *d* = 0.42, Pandey et al., 2018). Interestingly, Friese et al. (2017) showed that training effects were significantly larger when the task showing the training effect was preceded by a depleting effortful task (*g* = 0.60) rather than when it was not (*g* = 0.21). This last result suggests that benefits from self-control training are more pronounced for the capacity to maintain effortful control over time (i.e., stamina or resistance to cognitive fatigue) rather than the capacity to exert strong effortful control during a short period of time (i.e., strength of effortful control).

In young adults, the interventions included a large variety of training tasks, such as using a non-dominant hand, maintaining good posture, avoiding sweets, performing inhibitory control tasks (e.g., Stroop task) or practicing physical exercises. In preschool and kindergarten children, half of the interventions used a curriculum-based approach implemented in classrooms including circle-time games, storytelling, book reading, and self-talk. In preadolescents and adolescents, the training strategies mainly included activities such as role-playing, cognitive modeling, psychoeducational group therapeutic lessons, physical exercises, and mindfulness and/or yoga exercises. Nevertheless, the amount of effortful control required by this large diversity of activities is rarely assessed.

Regarding the transferability of gains in self-control, intervention studies with children and adolescents showed a main positive effect on far-transfer outcomes, such as academic achievement, mental health, social skills, frequency of school suspensions, and educational attainment, but a weaker effect on substance abuse when comparing the treatment group with the control group. In young adults, the effect of self-control training on far-transfer outcomes was not conclusive. The two most recent meta-analyses showed contradictory results. The meta-analysis of Beames et al. (2018) found that the effect sizes for health and well-being outcomes were small-to-medium and significantly different from zero whereas the meta-analysis of Friese et al. (2017) failed to show significant effects for the same outcomes.

### What Did We Learn From These Meta-Analytic Reviews?

In the present umbrella review of meta-analytic reviews, we analyzed the results from 63 meta-analyses interested in the effect of miscellaneous interventions aiming to durably improve EFs and self-control efficiency. A large majority of these meta-analyses (i.e., 79.37%, 50/63) showed that training programs are effective in improving performance in tasks tapping EFs and/or self-control with a small to large effect size. The transferability of these gains is more nuanced. Process-based and video game interventions failed to show far-transfer effects on academic or everyday functioning tasks. By contrast, self-control interventions seem more effective in producing far-transfer gains in other domains of self-control than trained domain. Intervention studies based on physical training listed in Table 2 and those based on mindfulness exercises rarely assess secondary outcomes, such as performance in academic or everyday functioning tasks. Consequently, it is difficult to assess the generalizability of these two types of interventions in the different domains of self-control. However, training effortful control through physical exercises or mindfulness exercises and observing gains in EFs could be considered far-transfer effects.

The interventions listed in the 63 meta-analyses mainly focused on children, adolescents and older adults, with the exception of mindfulness-based interventions. These three populations share a common characteristic: their EFs undergo drastic and quick changes in efficiency. Indeed, EFs are still developing in children and adolescents (De Luca et al., 2003; Blakemore and Choudhury, 2006) and declining in older adults (Spreng et al., 2017). Consequently, these populations situated at the two extremes of the lifespan are likely more sensitive to the effects of moderators, such as training and chronic stress, which improve or impair these high-level cognitive functions, respectively. For that reason, researchers should focus on these three populations when examining the effects of training on EFs and effortful control, because they would increase the likelihood to observe a significant effect.

For the same reason, it would be very interesting to examine the sensitivity to training for different symptomatic populations suffering from a recurrent mental fatigue (e.g., fragile older adults, multiple sclerosis patients, traumatic brain injured people or cancer patients treated with chemotherapy) or having a low dispositional capacity to exert effortful control (e.g., individuals with addictions, depression, obsessive-compulsive disorder or attention-deficit hyperactivity disorder). Few intervention studies targeting effortful control have been conducted in these populations.

If gains in EFs and self-control through training programs are based on durable changes taking place within large-scale networks, we can hypothesize that the stability of improvement in EFs and/or self-control over time could be an important index of training success. Consequently, intervention studies assessing near- and far-transfer effects in several follow-up assessments after program cessation are very good arguments for real durable changes.

Process-based cognitive interventions reported follow-up measurements in only 26.4% of the studies, whereas self-control, physical exercise, video game and mindfulness interventions rarely reported this type of information. The duration between the postintervention and the follow-up varied greatly among the studies reporting a follow-up: from 3 weeks to 10 years. When reported, effects on follow-up outcomes were significant with small to moderate size (Rapport et al., 2013; Nguyen et al., 2019; Takacs and Kassai, 2019), or non-significant (Lampit et al., 2019). However, several confounding factors, such as regular effortful activities practiced by participants in continuation of the training program or completely independent of the training program (e.g., playing chess outside of engaging with an aerobic exercise program), can moderate the outcomes associated with self-control and EF efficiency that are measured at follow-up, and these must be more rigorously controlled for in the future.

The quality of the 63 selected meta-analyses (see section S5 in Supplementary Material) is globally low. According to the AMSTAR2 risk of bias assessment scale (Shea et al., 2017), 54 meta-analyses (85.71%) are of critically low quality (*M* = 10.48/16; SD = 2.17), i.e., present more than one critical weakness. The three more frequent critical flaws are: (a) not providing a list of excluded studies with reasons of exclusion (87.30%), (b) not pre-registering the review methods prior to the conduct of the review (71.43%) and (c) not accounting for risk of bias in individual studies when discussing the results of the review (65.08%). Future meta-analyses on this topic will have to address these issues. However, a majority of the selected meta-analyses used a satisfactory technique for assessing the risk of bias in individual studies (83.13%), provided a satisfactory explanation for the heterogeneity observed in the results of the review (79.37%) and carried out an adequate investigation of publication bias with a discussion of its likely impact on the results of the review (77.78%).

This section clearly shows that all the above-mentioned training methods allow improving EFs and strengthening self-control. The generalizability of these gains seems more evident and robust in self-control training interventions. Which mechanisms can explain these gains and their transferability? The aim of the next section is to propose plausible and rational neurobiological mechanisms to explain the effects of effortful control training. A recent meta-analysis on the topic mentions that the mechanisms underlying these effects are poorly understood (Friese et al., 2017).

## Neural Bases of Gains in Effortful Control Capacity Through Training

The aim of this section is to clarify the neurophysiological mechanisms underpinning the improvements in effortful control capacity through training programs. We assume that the improvements in the capacity to exert effortful control results in learning processes based on long-term synaptic plasticity, which take place in specific regions of the central nervous system involved in the engagement of effortful control. The description of these mechanisms requires the use of a theoretical framework proposing several neuronal networks as possible targets of these durable changes in activity and/or connectivity with training. We will use the integrative model of effortful control proposed by André et al. (2019) as a model of reference.

According to this model, effortful control is a top-down oscillatory control signal generated by a large functional neuronal network called the salience network (Seeley et al., 2007; Seeley, 2019). Converging empirical evidence from neuroscience suggests that different brain structures involved in the salience network, such as the dorsal anterior cingulate cortex, integrate costs and benefits associated with the achievement of the ongoing task to make decisions about the amount of effortful control dedicated to this task (e.g., Kennerley et al., 2006; Shenhav et al., 2013, 2017; Klein-Flügge et al., 2016).

On the one hand, benefits are the immediate or delayed positive consequences associated with the achievement of the task goal. They include all types of rewards (e.g., food, money, pleasure, social rank). On the other hand, costs are associated with the detrimental consequences an individual has to cope with while attempting to achieve an intended goal, such as expending limited resources or feeling pain. They depend on task constraints (i.e., the higher the constraints are, the higher the costs are) and participant characteristics (i.e., the lower the capacity to exert effortful control is, the higher the cost of effort). They include different categories of costs that are detailed hereafter and summarized in Table 3.

**TABLE 3:** The different categories of costs that influence effort-based decision making and determine the amount of effortful control dedicated to a task.

Category of cost	Short definition
Metabolic or energetic costs	Muscular and brain glucose expended to reach the task goal
Computational costs	Number of processing-units recruited to perform a specific task regarding the finite number of available processing units
Motor costs	Energetic and computational costs associated with the performance of a movement or a motor skill; they involve muscular and brain costs
Executive control costs	Energetic and computational costs associated with the performance of a task requiring executive control; i.e., related to the processing units devoted to executive control
Risk-related costs	Costs associated with the risk of not obtaining a reward, losing an already obtained reward, or experiencing negative consequences while obtaining a reward
Pain-related costs	Costs associated with the risk of experiencing pain while attempting to reach a goal
Opportunity costs	This term was introduced by Kurzban et al. (2013). It designates costs associated with the engagement of the effort system to perform an effort-demanding task that prevents to perform other effort-demanding tasks
Intrinsic costs	This term was introduced by Shenhav et al. (2017). It designates costs associated with the exertion of effortful control, a capacity-limited function; i.e., energetic and computational costs related to effort-dedicated processing units

**FIGURE 1:**
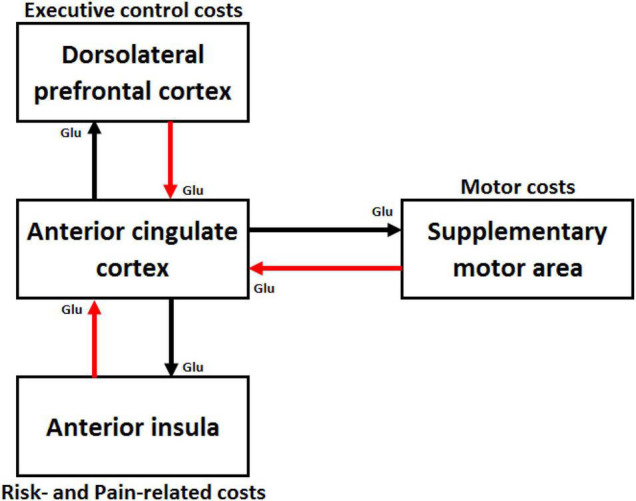
Schematic illustration of cortical areas involved in effort costs computation. The supplementary motor area is involved in computation of motor costs, the dorsal lateral prefrontal cortex in executive control costs, and the anterior insula in risk- and pain-related costs. These three regions are interconnected to the anterior cingulate cortex (ACC) through glutamatergic (Glu) pathways. The red arrows represent the cost signals sent by these cortical areas to the ACC that integrates costs and benefits signals and makes decisions on how much effort deploying to achieve the task goal. The black arrows represent the control signal sent by the ACC to the brain areas computing the cost signals to enhance their capacity of processing.

André et al. (2019) distinguished metabolic or energetic costs (e.g., muscular and brain glucose necessary to reach the task goal) and computational costs (e.g., number of effort-dedicated processing units devoted to the task). However, three other main categories of cost computed by different cortical areas have been described in neuroscience (see Figure 1).

The first and certainly most studied category includes costs related to the physical activity necessary to achieve the task goal. These motor costs encompass energetic costs associated with energy expenses made by the muscles (i.e., intensity of muscle contraction) as well as computational costs associated with the complexity of the movement (e.g., number of motor units involved, complexity of the coordination timing between these motor units). Several fMRI and transcranial magnetic stimulation (TMS) studies conducted in humans have suggested that the supplementary motor area (SMA) is involved in the coding of these motor costs (Pessiglione et al., 2007; Kurniawan et al., 2010; Burke et al., 2013; Zénon et al., 2015; Bonnelle et al., 2016).

The second category of costs is related to the degree of engagement of brain regions subserving EFs, such as working memory updating, inhibitory control and planning (Duncan and Owen, 2000; McGuire and Botvinick, 2010; Baumgartner et al., 2011). These executive control costs encompass energetic costs (i.e., brain glucose expended by each processing unit involved in executive control) and computational costs (i.e., number of processing units allocated to task performance relative to the limited number of available processing units). The dorsolateral prefrontal cortex (DLPFC), which lies in the middle frontal gyrus, is an important hub in the executive control network (Menon, 2011) and its activity is associated with the executive control costs. For instance, several fMRI and functional near-infrared spectroscopy (fNIRS) studies have shown that activation in the left DLPFC scales linearly with working memory load (Barch et al., 1997; Braver et al., 1997; Jansma et al., 2000; Veltman et al., 2003; Fishburn et al., 2014; McKendrick and Harwood, 2019), indicating load-dependent recruitment of the DLPFC. In addition, transcranial direct current stimulation of the left DLPFC, which facilitates neural activity within this cortical area, reduces the cost of performing a cognitive task on gait and postural control (Zhou et al., 2014). Finally, a more recent study showed that executive control costs are anticipated by the DLPFC (Vassena et al., 2019). Other cortical areas, such as the ventrolateral prefrontal cortex (VLPFC), which is located in the inferior frontal gyrus and ensures inhibitory control (Aron, 2007; Berkman et al., 2009; Aron et al., 2014a,b), could also participate to the computation in executive control costs.

The third and last category of costs includes both risk- and pain-related costs. Three types of risk-related costs have been identified: (1) the risk of not obtaining a reward, (2) the risk of losing an already obtained reward, and (3) the risk of experiencing negative consequences while obtaining a reward. A large number of studies have shown that the anterior insula computes these three types of risk (Burke and Tobler, 2011; Burke et al., 2013; for a meta-analysis, Mohr et al., 2010). This brain region is also involved in the subjective value of pain in effort-based decision-making (Talmi et al., 2009).

In this cost-benefit effort-based decision-making framework, two main mechanisms can explain a durable improvement in the capacity to exert effortful control with training: (1) a durable decrease in the effort costs; and (2) a durable increase in the value of the benefit resulting from goal-directed activities that requires effortful control (i.e., effort valuing). In the next subsections, we more precisely describe the two mechanisms that may underpin gains in effortful control capacity through exercise as well as mindfulness and self-control training.

### Durable Reductions in Effort Costs Through Physical Training

According to the first mechanism, regularly practicing effortful exercises would lead to a progressive reduction in effort costs: that is, practice increases efficiency, and makes better performance possible with the same or less effort. Motor costs, executive control costs and pain-related costs are likely to decrease with physical training.

Reductions in effort costs are frequently observed in kinesiology and sport sciences with regard to physical effort. It is easy to understand this common phenomenon: individuals who take part in a physical training program that includes effortful exercises generally improve cardiorespiratory fitness as well as muscular strength, and they become increasingly efficient at practicing these exercises (Lin et al., 2015; Montero and Lundby, 2015; Lee and Stone, 2020). Consequently, the same exercise (i.e., same duration and same absolute intensity) requires more effort and energy at the beginning of the training program than at its end. Perceived exertion decreases with training (e.g., Farhat et al., 2015). In this way, sedentary or physically unfit people who start regular exercises progressively develop a higher tolerance for exercise and effort (e.g., Gomes-Neto et al., 2016). Symmetrically, people with a high cardiorespiratory fitness perceive a given absolute intensity of exercise as less effortful than people with a low cardiorespiratory fitness do (Eston and Brodie, 1986; Pfeiffer et al., 2002).

In addition, if the gain in effortful control acquired through physical training is transferable to the cognitive domain, it can be inferred that this gain in efficiency should be observed in the activation of brain areas involved in tasks tapping EFs. More precisely, a decrease of activation in brain areas involved in the salience and/or executive control networks should be observed after the end of the physical training program compared to before its beginning.

A set of six studies explored the effects of chronic exercise on gains in executive control and their brain activation correlates. The researchers used a flanker task (Colcombe et al., 2004; Voelcker-Rehage et al., 2011; Chaddock-Heyman et al., 2013; Krafft et al., 2014), an antisaccade task (Davis et al., 2011; Krafft et al., 2014), or an n-back task (Nishiguchi et al., 2015) during fMRI scans before and after the exercise program. Half of the studies involved children, and the other half involved older adults. The duration of exercise programs varied from 13 weeks to 12 months. Four studies showed a positive effect of chronic exercise on behavioral performance, but two studies failed to find such an effect (Krafft et al., 2014; Nishiguchi et al., 2015). In contrast, all six studies showed a decrease in brain activity during the cognitive task at the end of the training program compared to the beginning, suggesting a higher efficiency in brain areas belonging to the salience network or the executive control network. These areas included the right dorsolateral prefrontal cortex (Voelcker-Rehage et al., 2011; Chaddock-Heyman et al., 2013; Nishiguchi et al., 2015), anterior cingulate cortex (Colcombe et al., 2004; Voelcker-Rehage et al., 2011; Krafft et al., 2014), posterior parietal cortex (Davis et al., 2011; Krafft et al., 2014), and right superior temporal gyrus (Voelcker-Rehage et al., 2011). These results suggested that physical training reduces the executive control costs associated with the performance of a cognitive task tapping EFs. In functional brain imagery, a decrease in BOLD response or blood flow in a specific brain region involved in the performance of the task and associated with a stable or better level of performance with repetition of the same task is generally interpreted as an increase in efficiency of the neuronal networks thanks to practice. In the present case, it would be a decrease in the need for top-down control and then a decrease in energetic cost associated with a lower top-down control. To our knowledge, no study examined the effect of chronic exercises on motor costs, i.e., BOLD fMRI variations, while performing a physical exercise before and after a physical training program, certainly because of the higher risk of head movement artifacts in the MRI scanner.

### Durable Reductions in Effort Costs Through Extensive Practice of Motor and Cognitive Skills

A decrease in computational cost, also known as attentional cost, can be observed with learning through a process of automatization. When people repeatedly perform a motor skill or a cognitive task, they progressively reduce the computational cost of the activity. From this perspective, the acquisition of automaticity can be viewed as the gradual withdrawal of effortful control. A large number of studies using the dual-task protocol have supported the fact that throughout the process of motor skill acquisition, the involvement of effortful control (i.e., attentional control) decreases across training sessions or blocks of trials (e.g., Brown and Carr, 1989; Wulf et al., 2001; Goh et al., 2014). The tenet of these studies is that the lower the attentional cost of performing the primary task (i.e., the motor skill) while simultaneously carrying out the secondary task (i.e., a cognitive task tapping executive control) is, the higher the automaticity of the motor skill.

This reduction in computational cost can be explained within the framework of the integrative model of effortful control. As mentioned earlier, this model assumes that effort is a mechanism anchored in a large functional neuronal network called the salience network (Seeley, 2019). The ‘mechanism of effort’ includes a limited number of interconnected processing units that integrates information regarding the costs and benefits associated with the achievement of the task goal and generates the effort signal, which is a top-down control signal optimizing the information processing of miscellaneous brain regions involved in the task. These effort-dedicated processing units are assumed to be anchored in the cortical minicolumns belonging to several cortical areas in the salience network, such as the anterior cingulate cortex, frontal operculum and anterior insula.

A high engagement of effortful control in the initial phase of learning followed by a progressive decrease in the need for effortful control in later phases of learning should be observed at the level of effort-dedicated processing units. Two hypothetical complementary mechanisms can explain this reduction in effortful control with learning: (1) the recruitment of a lower number of effort-dedicated processing units to perform the task and/or (2) a higher efficiency of these processing units at exerting effortful control (i.e., strengthened connectivity within each processing unit). These two mechanisms should lead to a lower activation of brain regions belonging to the salience network, and other top-down control brain regions involved in the task, such as the executive control network, by the end of the acquisition phase. Overall, fMRI studies examining patterns of activation in brain areas during cognitive tasks support quite well the hypothesis of a decrease in energetic and/or computational costs following several weeks of a training program that could include cognitive tasks or motor skills.

A set of six intervention studies confirmed that process-based cognitive training and motor skill learning led to a decrease in activation in brain areas belonging to the salience and executive control networks. The authors of these studies asked their participants to practice the following tasks: a self-initiated, self-paced, memorized sequential finger motor task while performing a letter-counting task (Wu et al., 2004); a visual serial reaction time task while performing a tone-counting task (Poldrack et al., 2005); an emotion regulation task (Berkman et al., 2014); a stop-signal task involving motor response inhibition (Beauchamp et al., 2016); and an n-back task (Heinzel et al., 2016; Miró-Padilla et al., 2019). The training volume ranged from 60 min (Berkman et al., 2014; Beauchamp et al., 2016) to 540 min (Heinzel et al., 2016), and participants were mainly young adults except for one study that preferentially included older adults (Heinzel et al., 2016). The results of these six studies confirmed a decrease in BOLD activity in brain regions in the salience network (e.g., anterior cingulate cortex) but also in numerous other regions in the executive control network, such as the dorsolateral prefrontal cortex confirming a decrease in executive control cost with training.

### Increase in Connectivity: A Biomarker of Efficiency

An increase in the efficiency of effort-based processing units reflecting task automatization should also be evidenced by an increase in connectivity within the salience network and/or between the salience network and other large-scale networks, such as the executive control network: the higher the between-network connectivity is, the lower the effort cost. As mentioned earlier, these changes in connectivity are generally observed by using resting-state fMRI coupled with a seed-based functional connectivity analysis.

We found five studies using this method that focused on the link between gains in automaticity or performance through process-based cognitive training and an increase in connectivity within and between top-down control networks. First, Mohr et al. (2015) showed that a higher connectivity between the salience network and the dorsal attention network correlated with practice-related efficiency gains. These authors also observed that short-term task automatization was accompanied by decreased activation in the executive control network, indicating a release of high-level cognitive control, and a segregation of the default mode network from task-related networks. Second, Chapman et al. (2015) conducted a 12-week gist reasoning training and observed that functional connectivity increased monotonically within the default mode and executive control networks, from pre-training to the end of training and from pretraining to midtraining, respectively, in the process-based cognitive training group relative to the control group. Third, Cao et al. (2016) examined training-related changes in functional connectivity within and between the default mode, executive control and salience networks 1 year after the training ended. In their experiment, healthy older adults were randomly included in a 3-month multidomain process-based cognitive training group or in a wait-list control group. The authors observed increased functional connectivity within the executive control network after training compared with the baseline. Fourth, Thompson et al. (2016) examined functional connectivity within and between the executive control and dorsal attention networks in young adults during task performance before and after 20 days of training on either a dual n-back working memory task or a demanding visuospatial attention task involving multiple object tracking. Learning selectively occurred in the n-back training group, who displayed marked gains on the trained task and not in the visuospatial attention training group. This n-back training induced significant increases in functional connectivity within and between the two networks. Fifth, Sánchez-Pérez et al. (2019) showed that a computer-based program aiming to train schoolchildren in cognitive tasks that mainly tap working memory leads to improvements in cognitive and academic skills compared with an active control group. They also found stronger relationships between inhibitory control scores and functional connectivity within the executive control network in trained children than in children from the control group.

In light of all the results presented in the two preceding sections, we can conclude that the hypothesis of a decrease in effort costs with training is plausible and supported by behavioral as well as activation and resting-state functional brain imaging data.

### Durable Increases in Effort Valuing With Training

According to the second mechanism, prolonged experience in exerting effortful control would increase the value of a goal that required effort to be reached (e.g., Inzlicht et al., 2018): the higher the level of practice in effortful tasks is, the higher the expected benefit from any activity that requires effortful control. This hypothesis was initially formulated by Eisenberger in the framework of learned industriousness theory (Eisenberger et al., 1976; Eisenberger, 1992). This theory is based on the operant conditioning process (Skinner, 1938), a type of associative learning process through which the strength of a behavior is modified by a reinforcer. In operant conditioning, reinforcement occurs only after the organism intentionally executes a specified behavioral act. For instance, a child may learn to perform a chore without complaints to receive praise. From this perspective, animals and humans learn to engage in effortful tasks to maximize rewards. The learned industriousness theory views effort as a secondary reinforcer. If an organism learns that effortful tasks are consistently associated with greater rewards, the feeling of effort experienced during a task increases the expectation of a large reward once the task is performed.

Robert Eisenberger and his team conducted a series of intervention studies in animals and humans from the seventies to the nineties to demonstrate the soundness of this theory. The first experiment included a training program staggered over several days and was conducted with children (Eisenberger et al., 1985). In this experiment, 46 children were separated into three groups. Participants in the first group were paid for high effort in tasks involving object counting, picture memory, and shape matching, whereas participants in the second group were paid the same amount of money for a low-effort version of the same tasks. Participants in the third group did not undergo effort training. The training program for the first two groups included three training sessions given on consecutive days. Before and after the training program, all the participants made repeated choices between the tedious tasks of copying non-sense words for a large monetary reward versus waiting the equivalent duration for a small monetary reward. Before the intervention, the three groups did not differ in the number of times they chose to work for the larger reward. By contrast, after the intervention, the high-effort group chose the high-effort/high-reward alternative more frequently than did either the low-effort group or the control group, whereas the latter two groups did not significantly differ. Eisenberger and Adornetto (1986) replicated these results in a very similar experiment that manipulated the delay to the reward in addition to the effort required to obtain the reward. The results of these two studies clearly showed that repeatedly rewarding high levels of effort increases a person’s generalized choice of high-effort large rewards over low-effort small rewards and may contribute to individual differences in the willingness to postpone gratification in pursuit of long-term goals.

In a third experiment, Eisenberger et al. (1989) replicated these results in animals and trained two groups of rats to run down a runway for food pellets in a low-effort or high-effort condition for 18 days. In the low-effort condition, the rats received one pellet for one trip during the entire training period, whereas in the high-effort condition, they received one pellet for one trip at the beginning of training and one pellet for five trips at the end of training. Two groups of rats were added as control groups and received the same number and temporal distribution of pellet presentations as in the two experimental groups but without the instrumental requirement (i.e., completion of a given number of round trips). At the end of the training program, the four groups of rats performed 12 choice test sessions the same day. They were tested by giving repeated choices of exerting low force on one lever for a small reward versus exerting high force on the alternative lever for a large reward. The results clearly showed that rats in the high-effort training group chose the high-effort, large-reward goal box more frequently than the three other groups. These results demonstrated that training animals in a rewarding high-effort task during several sessions increased the likelihood that these animals chose to exert a higher level of effortful control associated with a higher reward in a subsequent transfer task. A more recent study (Laurence et al., 2015) replicated these results in rats with a similar protocol but with a longer training program. For a period of 7 weeks, exercise rats were individually placed in a rodent running ball for five sessions per week (20 min/session). To our knowledge, this series of experiments initiated by Eisenberger constitutes the first elements of proof that repeatedly associating high effort with high reward during a training phase can transfer to other tasks and drive the trained individuals to choose more effortful tasks to increase the likelihood of gaining more benefits.

**FIGURE 2:**
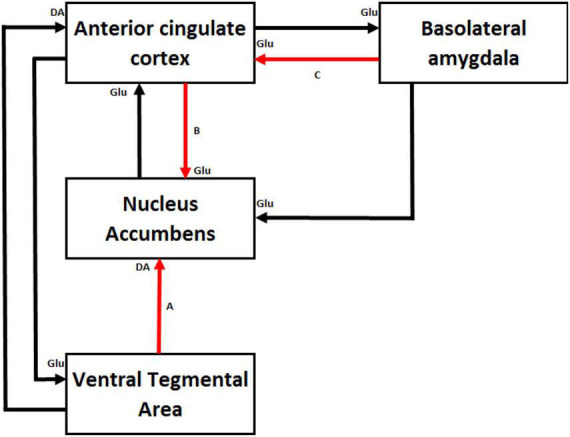
Schematic illustration of the key structures and neurotransmitter pathways involved in effort-based decision-making in rodents and more particularly those that allow animals to overcome effort costs to obtain higher rewards. Pathway A connects the ventral tegmental area to the nucleus accumbens (NAC). Pathway B connects the anterior cingulate cortex (ACC) to the NAC. Pathway C connects the basolateral amygdala (BLA) to the ACC. Destruction of dopamine terminals in the NAC (Cousins and Salamone, 1994), lesions of the ACC (Walton et al., 2002) and bilateral inactivation of the BLA (Floresco and Ghods-Sharifi, 2007) impair effort-based decision-making and reduce the preference of animals to exert more effort to obtain a larger reward. These three structures clearly participate to a bias of behavior toward response options leading to larger rewards that come at larger costs but their respective contribution differ. In situations where an animal must choose between response options associated with differential magnitudes of reward, BLA neurons would encode the expected magnitude of reward that each choice may provide. This reward-related information would be relayed to the ACC *via* glutamatergic (Glu) projections. The ACC would bias behavior in a particular direction by integrating these reward-related signals with other information about response costs associated with each action. Then, the ACC would send the result of the decision-making to the NAC for an implementation of the appropriate behavioral output. Dopaminergic (DA) input from the ventral tegmental area to the NAC would be essential to energize appropriately the chosen instrumental activity in order to obtain the expected reward.

Where do the long-term synaptic changes underpinning the association between high effort and high reward take place in the brain? A series of experiments mainly conducted in rodents identified a set of four interconnected key structures allowing animals to overcome effort costs to obtain greater benefits. Figure 2 illustrates the connections between these four structures: the anterior cingulate cortex (ACC), nucleus accumbens (NAC), basolateral amygdala (BLA), and ventral tegmental area (VTA).

John Salamone from the University of Connecticut and his collaborators took a first step in the comprehension of effort-based decision-making. In rodents, effort-based decision-making is typically assessed using tasks that offer animals a choice between a relatively preferred reinforcer (i.e., reward) that can only be obtained by a high exertion of effort versus a lower effort/lower value option (for reviews, see Assadi et al., 2009; Salamone et al., 2018). In the first experiment, working for a preferred food (i.e., high carbohydrate pellets) by lever pressing was the high-effort/high reward option, whereas simply approaching and consuming a less rewarding food (i.e., ordinary lab chow) was the low-effort/low-reward option (Cousins and Salamone, 1994). Rats typically pressed at high rates to obtain the preferred food and ate little of the lab chow; i.e., they preferentially chose the high-effort/high-reward option. However, dopamine depletions produced by injections of the neurotoxic agent 6-hydroxydopamine (6-OHDA) in the NAC produced a dramatic decrease in lever pressing and an increase in chow consumption (Cousins and Salamone, 1994).

These results have been replicated in a different experimental setup (Salamone et al., 1994; Cousins et al., 1996; Denk et al., 2005). Rats were trained on a T-maze task with one arm containing a large reinforcer (four pellets) associated with a large vertical barrier (44 cm) and the other arm containing a small reinforcer (two pellets) associated with unobstructed access. Similar to previous experiments, in standard conditions, animals prioritized the high-effort/high-reward option, and this effect was reversed when 6-OHDA was injected into the NAC or when rats received injections of 0.1 mg/kg haloperidol, a dopamine antagonist. In other words, disruption of the dopaminergic pathway by drug treatment led rats to prefer the low effort/low reward option. These results showed that across a wide variety of tasks, administration of low doses of DA antagonists and NAC DA depletions have a detrimental effect on effort-based decision-making, producing a low-effort bias that shifts animals away from the high-effort option and toward the low-effort choice. Other authors obtained similar results with similar experimental setups and different dopamine receptor antagonists, such as flupenthixol (Floresco et al., 2008). A similar paradigm in which subjects choose between two options with different benefits and costs and a manipulation of dopamine availability has not yet been tested in primates or humans (Assadi et al., 2009).

Mark Walton from the University of Oxford and his collaborators used the same paradigm but targeted the ACC (Walton et al., 2002, 2003, 2009; Rudebeck et al., 2006, experiment 2). As Salamone and his team showed, all animals preferred to select the high-cost/high-reward option in the standard T-maze task. In these experiments, rats had to choose between a high effortful action (i.e., climbing a 30-cm barrier) to obtain a large quantity of reward (high-cost/high-reward) or a lower effortful action (i.e., climbing a 10-cm barrier) to obtain a smaller reward (low-cost/low-reward). However, after excitotoxic lesions of the ACC, rats selected the low-cost/low-reward response on nearly every trial. In contrast, both control animals and rats with prelimbic and infralimbic lesions continued to choose to climb the larger barrier for the larger reward. These results indicated that the ACC is an important region within the medial frontal cortex when evaluating how much effort to expend for a specific reward.

Stan Floresco from the University of British Columbia took a third step in the comprehension of brain mechanisms supporting effort-based decision-making (Floresco and Ghods-Sharifi, 2007). In their first experiment, they used exactly the same T-maze task as Walton and coworkers but focused on the role of the BLA in the effort-based decision-making process. They replicated the results in standard conditions and observed that bilateral inactivation of the BLA *via* infusion of the local anesthetic bupivacaine hydrochloride impaired decision-making by reducing the preference for the high-effort/high-reward arm.

From the above, we hypothesize that in animals and humans, the generalized bias toward high effort/large rewards resulting from effortful control training is inscribed within the circuitry described in Figure 2, and more specifically, in glutamatergic synapses connecting the BLA, ACC, NAC and VTA. To our knowledge, only one recent study conducted in humans with fMRI (Bernacer et al., 2019) showed that functional connectivity between the amygdala and ACC was strengthened after a 3-month fitness program (20–30 min sessions of walking and running on a treadmill, 2–3 days a week for 3 months).

The two preceding sections show that our field needs more theory-driven studies using animals as well as activation and resting-state fMRI in humans to determine precisely where when and how these durable changes in neural activity and connectivity occur. Some methodological suggestions in this direction will be made in the following section.

## Challenging the Trainability of Effortful Control Capacity

The preceding sections provide arguments for a possible strengthening of effortful control capacity through the practice of effortful tasks. Then, two plausible mechanisms have been proposed to explain these gains in effortful control capacity. The aim of this last section is to address several theoretical and methodological issues to improve the effectiveness of training programs and comprehension of the mechanisms that underpin these gains in effortful control capacity.

The first issue concerns the choice of an appropriate protocol to prove and generalize a causal relationship between the regular practice of effortful tasks and durable improvements in effortful control capacity. The best way to eliminate bias that comes from confounders and demonstrate causality is to conduct randomized controlled trials (RCTs). In RCTs, study participants are randomly assigned to either receive the treatment or be in a control group (placebo). In the present case, the treatment group receives the training program aiming to improve effortful control capacity.

Proposing a control intervention that is as similar as possible to the treatment intervention with the exception that the level of effortful control differs across group activities is certainly the most difficult methodological issue to address in the context of an RCT protocol using human activities. An appropriate strategy could be to include two control groups: an active control group practicing activities requiring little effort (e.g., relaxation exercises, passive stretching exercises, massage and hydromassage sessions, watching emotionally neutral but interesting documentaries) and a passive control group that does not change its life habits during the period of the intervention. Fifteen out the 63 meta-analyses included in the present systematic review considered the type of control group as a moderator of the effect size of the intervention. Five out of these 15 meta-analyses showed that the effect size was significantly larger for studies that used a passive control group rather than an active one (Karr et al., 2014; Beames et al., 2018; Northey et al., 2018; Nguyen et al., 2019; Ren et al., 2021).

Regarding the treatment intervention, we recommend the use of effortful exercises (e.g., a combination of aerobic and resistance exercises) that stimulate brain plasticity (Fernandes et al., 2017; Walsh and Tschakovsky, 2018), in combination with cognitive tasks tapping EFs or mindfulness exercises. Physical exercises and cognitive tasks can be performed sequentially or simultaneously (team games or situational problem-solving tasks). The same is true for physical exercises and mindfulness exercises (e.g., yoga).

The second issue concerns the content of the treatment intervention program to generate transferable gains in effortful control capacity. In this perspective, the training program must be tailored, progressive and varied to optimize the likelihood of success in obtaining the desired effect. Tailoring the program means individualizing task difficulty and exercise intensity (e.g., difficulty expressed in percentage of individual’s maximal capacity). The respect for this first principle ensures that there will be no large imbalances in perception of task-related constraints across participants, thereby resulting in quite similar levels of engagement. The second principle concerns the progressive increase in task difficulty and exercise intensity throughout the training program. This second principle allows the maintenance of a high level of participant engagement throughout the program. At last, it is important to vary training exercises to improve the generalizability/transferability of gains in the capacity to exert effortful control (Eisenberger et al., 1982) and reduce boredom.

The third issue concerns the choice of the outcomes that will assess the gain in effortful control capacity. These outcomes can be assessed at three levels of observation (i.e., subjective, behavioral, and physiological) and at different times of the intervention study (e.g., before and after the program). Behavioral indexes, such as the level of performance in a specific task, are valuable data that provide information about the level of engagement of the participant in the task and his/her skill level in this task. Experimenters need to choose tasks sensitive to practice effects with no risk of ceiling effects. The subjective measurements, such as effort required to perform the task and perceived fatigue at the end of the task, contribute to and facilitate the interpretation of results. Physiological indexes of effort engagement (i.e., effortful control), such as pupil size, pre-ejection period (PEP) and prefrontal theta power density, may contribute to the picture by adding objective measurements of effort costs and top-down control to cope with the task goals. All these indexes (subjective, behavioral, and physiological) are complementary and make their own contribution to understanding variations in outcomes as a function of the intervention. A large majority of RCTs selected in the reviewed meta-analyses did not use physiological indexes of effortful control.

In addition to the outcomes described previously, we recommend assessing at least three categories of transfer outcomes: (1) near-transfer outcomes such as performance in tasks tapping EFs and self-control (e.g., use of the sequential task protocol before and after the intervention); (2) far-transfer outcomes related to performance in everyday functioning tasks, such as academic performance; and (3) far-transfer outcomes concerning general self-regulation abilities, such as snacking, speeding, and periods of inattention. It could also be appropriate to have several follow-up assessments (or retention tests), e.g., 1 month, 3 months, and 6 months after the end of the training program, to show stability of the gains in effortful control capacity. Few interventional studies include follow-up measurements.

The fourth and last issue concerns the choice of an appropriate method that allows a better understanding of the durable changes in connectivity occurring within and between several large-scale neuronal networks involved in effortful tasks, such as the salience network, the executive control network, the default network and the mesolimbic network. In the future, resting-state and activation functional MRI techniques in conjunction with graph theory could be used before and after the training program to disentangle the role of these brain networks in the improvement to the capacity to exert effortful control. Only few interventional studies used functional MRI to assess network connectivity.

We are aware that the type of RCT described above is time and money consuming, but it is the best guarantee to demonstrate that this type of intervention is a plausible and possible way to train the effortful control capacity and explain which mechanisms underpin these durable gains. In addition, the gains provided by the identification of the determinants of the effectiveness of willpower training programs overcome the costs of the research leading to such scientific advances. As mentioned in the introduction, these gains in willpower can increase the likelihood of success, well-being and productivity of each individual in society.

## Conclusion

The first question we addressed in this paper concerns the existence of empirical evidence that supports possible gains in effortful control capacity through training. In the second section “Improvements in Effortful Control with Practice: An Umbrella Review of Meta-Analytic Reviews,” we provided clear evidence that executive control and effortful control can be improved through interventions using physical, cognitive or mindfulness exercises. However, we showed that the generalizability of these gains depends directly on the type of training interventions. In other words, people can definitely be trained to improve their executive functioning and self-control, but results have been inconsistent and variable as to how widely the improvements generalize to tasks different from those used in the training. Self-control training programs seem more effective than process-based training programs in inducing generalizability. Moreover, physical and mindfulness exercises seem to be two promising training methods that deserve to be included in self-control training programs. The higher effectiveness of self-control training programs in leading to generalizable gains most likely rests on the fact that these training programs include a greater variety of effortful tasks than process-based training programs.

The second question concerns the durable changes in brain structure and brain functioning that explain these increments in the capacity to exert effortful control. We pointed out two plausible brain mechanisms that can explain these gains in top-down control: (1) a decrease in effort costs combined with a greater efficiency of brain regions involved in the task and (2) a change in the value of effort through operant conditioning in the context of high effort and high reward. Our article shows that these two mechanisms have received clear empirical support from functional brain imaging studies in humans and neurophysiological studies in animals. The first mechanism is rather in favor of the hypothesis of the strengthening of the capacity to exert effortful control (i.e., more effortful control with less energy). By contrast, the second mechanism rather supports the motivational hypothesis: a durable predisposition to engage in effortful activities (i.e., an amplification of the benefit signal). Both mechanisms are certainly synergistic in contributing to how training improves effortful control. In addition, Bavelier and Green (2019) presented very interesting arguments suggesting that these two systems (i.e., the attentional/effortful control system and the reward system) foster learning and brain plasticity.

Based on the present literature review, what are the most pressing questions that would need further data collection on this topic in the near future? First, we need more resting-state electroencephalographic (EEG) and brain imaging studies examining the durable changes in connectivity, within and between large-scale neuronal networks, induced by training programs aiming to improve the capacity to exert effortful control. Three between-network connectivity hypotheses could be tested: (1) an increase of connectivity with training between the salience network and the executive control network supporting the strengthening hypothesis, (2) a decrease of connectivity with training between the executive control network and the default-mode network also supporting the strengthening hypothesis, and (3) an increase in connectivity between the mesolimbic reward network and the salience network supporting the motivational hypothesis. Second, we need to define more precisely the characteristics of the theory-based training programs that are more effective to strengthen the general capacity to exert effortful control, more particularly the most effective training exercises and the minimum volume of training needed to obtain significant gains according to the target population. Third, we need to know which theory-based behavioral change techniques are most effective at maintaining an effortful training program in the long-term.

Finally, if training programs are effective in strengthening effortful control capacity, citizens should be encouraged to practice and maintain engagement with such programs over the long term to continue developing these gains throughout their lives. In this way, health policies could promote the maintenance of a virtuous circle between healthy behaviors, including “willpower training” and the capacity to exert effortful control (for a description of this virtuous circle, see Audiffren and André, 2019). Based on this virtuous circle, training improves the capacity to exert effortful control and then a higher capacity to exert effortful control facilitates the maintenance of training and healthy behaviors.

## Author Contributions

MA, NA, and RB participated to the conceptualization of the theoretical ideas, writing of the article, and manuscript revision. MA and NA conducted the literature reviews. MA elaborated the tables and figures. All authors approved the submitted version.

## Conflict of Interest

The authors declare that the research was conducted in the absence of any commercial or financial relationships that could be construed as a potential conflict of interest.

## Publisher’s Note

All claims expressed in this article are solely those of the authors and do not necessarily represent those of their affiliated organizations, or those of the publisher, the editors and the reviewers. Any product that may be evaluated in this article, or claim that may be made by its manufacturer, is not guaranteed or endorsed by the publisher.
