# Supplementary material for: Training Willpower: Reducing Costs and Valuing Effort
Source: Front Neurosci. 2022 Apr 28;16:699817. doi: 10.3389/fnins.2022.699817 (PMC9095966; doi:10.3389/fnins.2022.699817)
Supplement: Supplementary file 1 [file Data_Sheet_1.pdf]

# Supplementary material

This supplementary section describes the methods used to conduct the different systematic reviews presented in the manuscript.

**S1. Search strategy**

The sources used to identify the meta-analyses targeted by the systematic reviews included Academic Search Complete, MEDLINE with full text, CINAHL with full text, APA Psycinfo, and SPORTDiscuss with full text through EBSCO host. The search covered articles published until November 30, 2021.

***S1.1. Search terms for the systematic review of meta-analyses concerning the effect of process-based cognitive training on executive functions***

(Cognitive N0 training OR Cognitive N0 intervention OR Cognitive N0 therapies – in subject terms OR title) AND (Working N0 memory OR Inhibitory control OR Cognitive N0 flexibility OR Executive N0 function* OR Cognitive N0 domain – in abstract OR subject terms) AND (meta-analy* OR meta-regression – in title).

***S1.2. Search terms for the systematic review of meta-analyses concerning the effect of exercise training on executive functions***

(Exercise OR Physical N0 activity OR Fitness OR Dance OR Tai Chi – in title) AND (Cognition OR Cognitive OR Neurocognitive OR Executive N0 function – in title) AND (meta-analy* OR meta-regression – in title)

***S1.3. Search terms for the systematic review of meta-analyses concerning the effect of interventions combining exercise and process-based cognitive training on executive functions***

(Exercise OR Physical – in title) AND (Cognitive – in title) AND (Combined OR enriched – in title) AND (Meta-analy* OR Meta-regression – in title).

***S1.4. Search terms for the systematic review of meta-analyses concerning the effect of video game training on executive functions***

(Video N0 game OR Exergames OR Serious N0 game– in title OR abstract) AND (Meta-analy* OR Meta-regression – in title OR abstract).

***S1.5. Search terms for the systematic review of meta-analyses examining the effect of mindfulness training on executive functions***

(Mindfulness OR Meditation– in title OR abstract) AND (Intervention OR Training OR Therapy OR Induction – in title OR abstract) AND (Meta-analy* OR Meta-regression – in title OR abstract).

***S1.7. Search terms for the systematic review of meta-analyses concerning the effect of self-control training***

(Self-control OR Self-regulation – in title OR abstract) AND (Practice OR Training OR Exercise OR Improvement OR Intervention* – in title OR abstract) AND (meta-analy* OR meta-regression – in title OR abstract)

**S2. Eligibility criteria**

***S2.1. Eligibility criteria common to all systematic reviews***

The search process focused on meta-analyses aiming to examine the effects of different types of intervention on executive functions or self-control. The search was limited to published articles written in English and reporting data from human participants. Unpublished meta-analyses were not included in the systematic reviews. The meta-analytic studies had to include at least eight randomized controlled trials (RCT) and report effect size for executive functions or self-control.

***S2.2. Eligibility criteria specific to the systematic review of meta-analyses concerning the effect of process-based cognitive training on executive functions***

Fifty articles were not included in the present systematic review for different reasons listed in Figure S1. The three main reasons were: (1) the meta-analysis did not focus on process-based cognitive training (16 meta-analyses); (2) the number of effect sizes reported for executive functions is less than 8 (15 meta-analyses); (3) no effect size were reported for executive functions (11 meta-analyses).

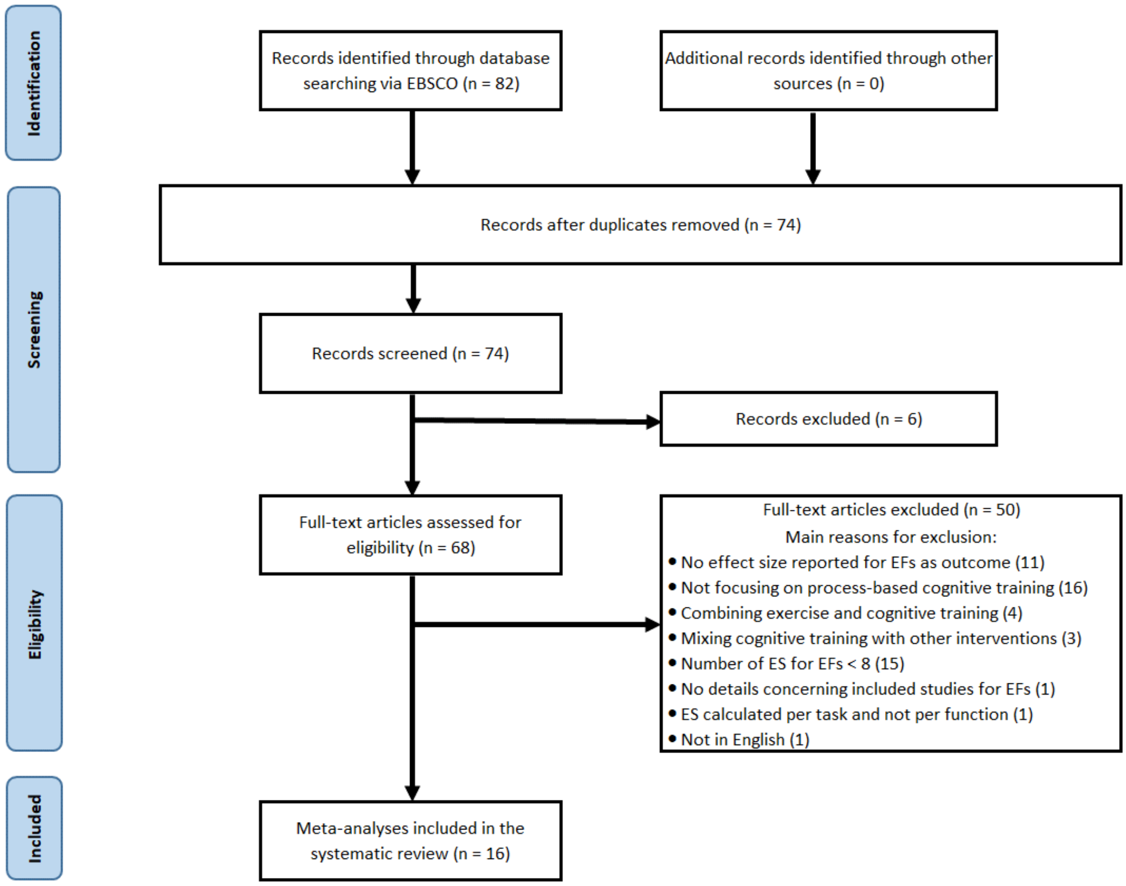


**Figure S1:** Flow chart of meta-analyses selected for the systematic review on the effect of process-based cognitive training on executive functions

***S2.3. Eligibility criteria specific to the systematic review of meta-analyses concerning the effect of exercise training on executive functions***

Meta-analyses mixing exercise training interventions with another type of intervention (e.g., cognitive training) or comparing exercise training to another type of treatment (e.g., nutrition interventions, exergames) were not selected. In the same way, meta-analyses focusing on acute exercise or mixing acute exercise and chronic exercise were not included in the review. Figure S2 describes the selection process of the systematic review of meta-analyses on the effect exercise training on executive functions.

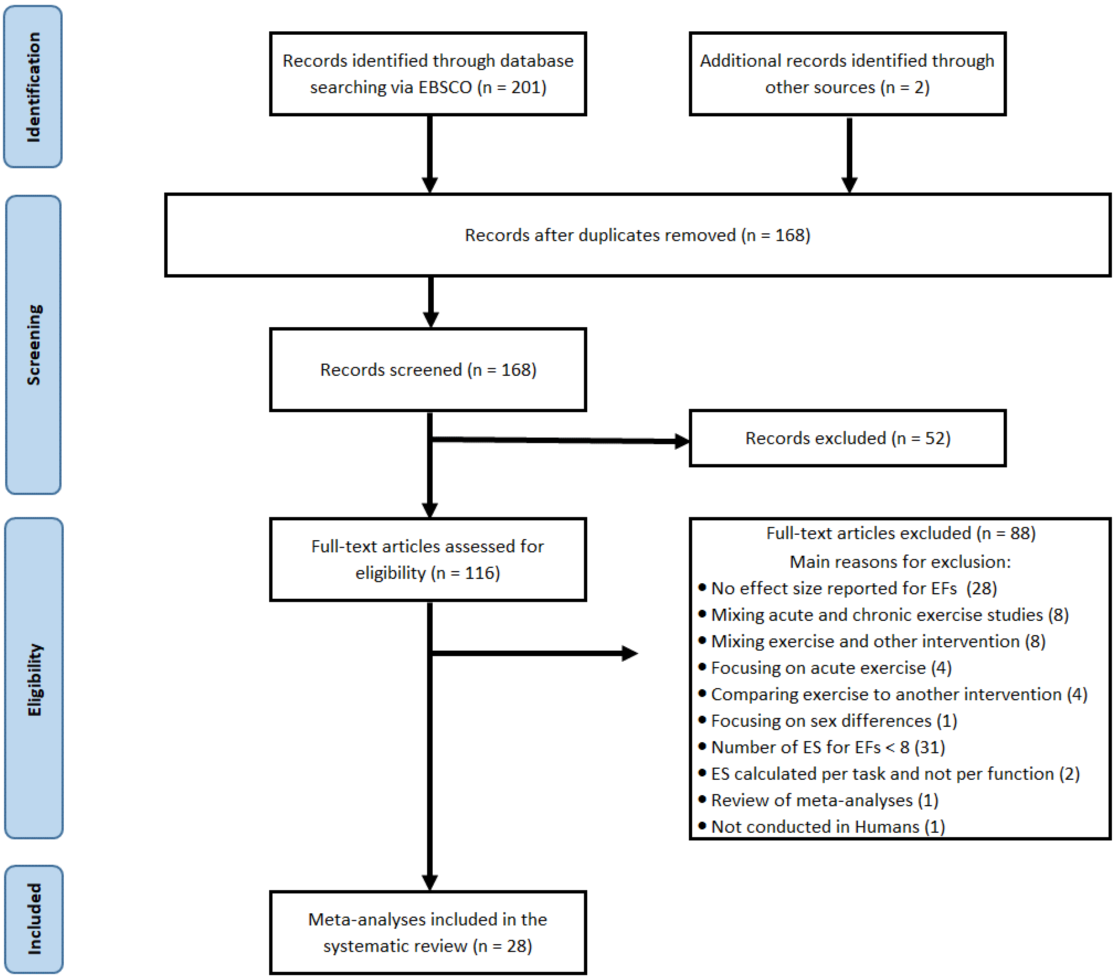


**Figure S2:** Flow chart of meta-analyses selected for the systematic review on the effect of exercise training on executive functions

***S2.4. Eligibility criteria specific to the systematic review of meta-analyses concerning the effect of interventions combining exercise and process-based cognitive training on executive functions***

Nine articles were not included in the systematic review for the different reasons listed in Figure S3.

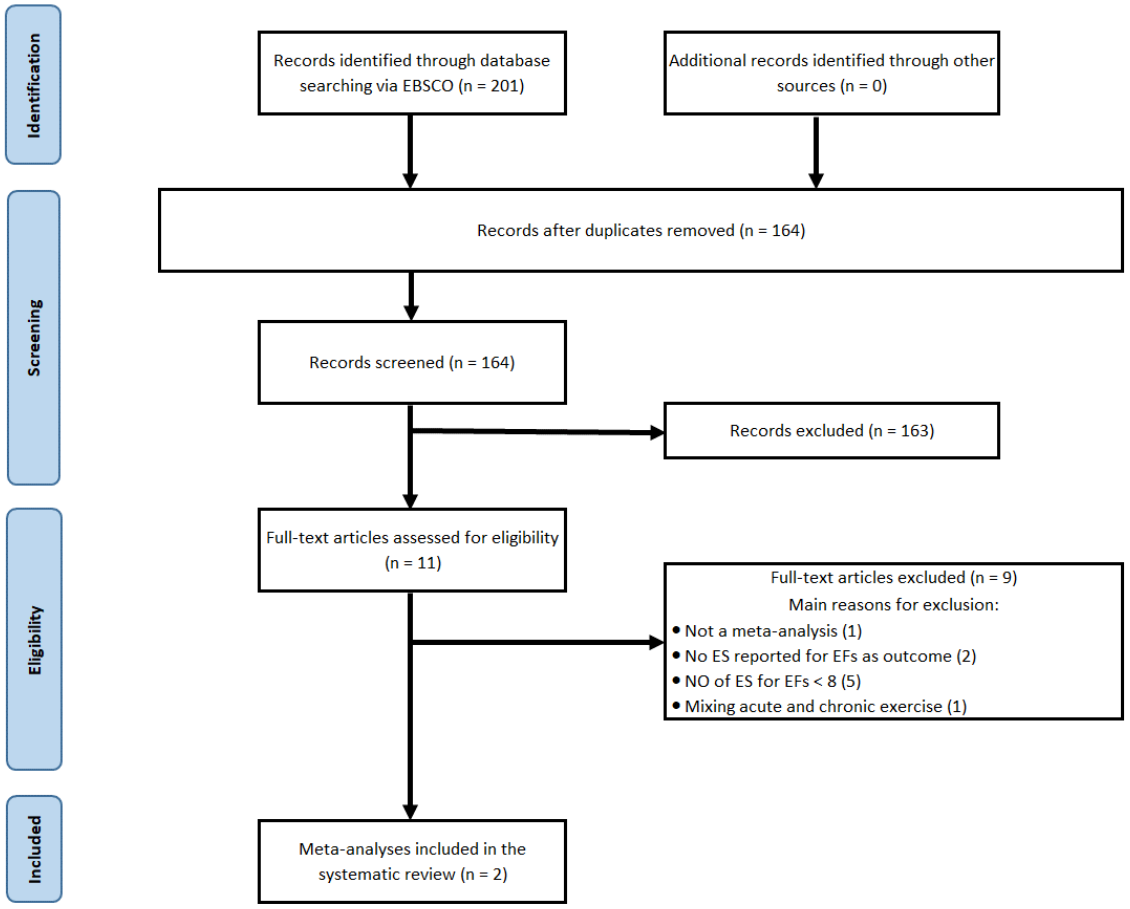


**Figure S3:** Flow chart of meta-analyses selected for the systematic review on the effect of interventions combining exercise and process-based cognitive training on executive functions

***S2.5. Eligibility criteria specific to the systematic review of meta-analyses concerning the effect of video game training on executive functions***

Nineteen articles were not included in the systematic review. Seven articles were systematic reviews instead of meta-analyses and two meta-analyses were not published (i.e., PhD dissertation). Two meta-analyses used less than 8 studies to calculate the effect size related to EF outcomes. Two meta-analyses did not report an effect size for EFs. Three meta-analyses mixed different categories of control groups when calculating effect sizes (e.g., control group practicing an effortful physical exercise and waiting list). Two meta-analyses included studies using a non-adequate research protocol (e.g., acute intervention instead of chronic intervention, cross-sectional instead of interventional study). Two meta-analyses were included in a non-published document (PhD or Master thesis). One meta-analysis did not mention the number and the references of interventional studies used to calculate

the effect sizes. Figure S4 describes the flow chart of the selection process for this set of meta-analyses.

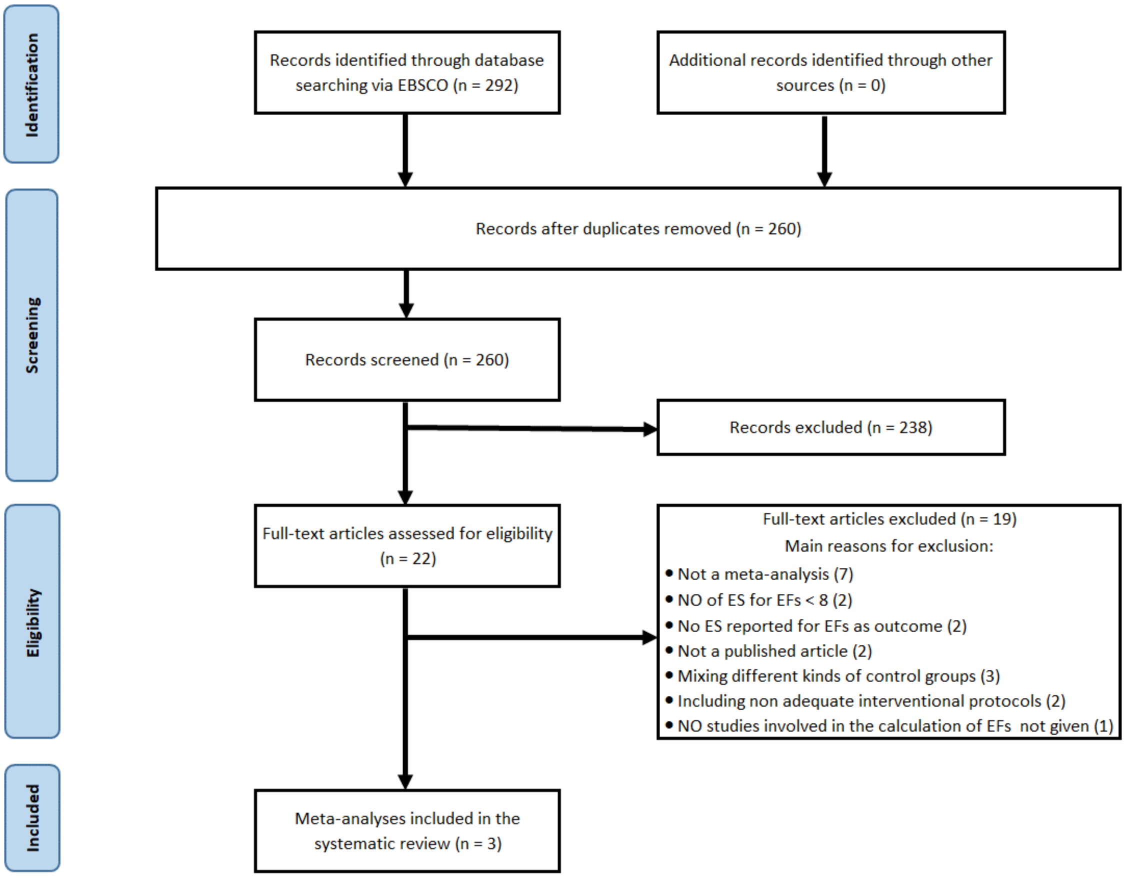


**Figure S4:** Flow chart of meta-analyses selected for the systematic review on the effect of video game training on executive functions

### ***S2.6. Eligibility criteria specific to the systematic review of meta-analyses concerning the effect of mindfulness training on executive functions***

Twenty articles were excluded from the systematic review. Twelve meta-analyses did not report an effect size for EFs. Three articles were not a meta-analysis of interventional study. Two meta-analyses used less than 8 studies to calculate the effect size related to EF outcomes. Three other meta-analyses focused on acute effects of mindfulness exercise or mixed acute and chronic effects of mindfulness exercise.

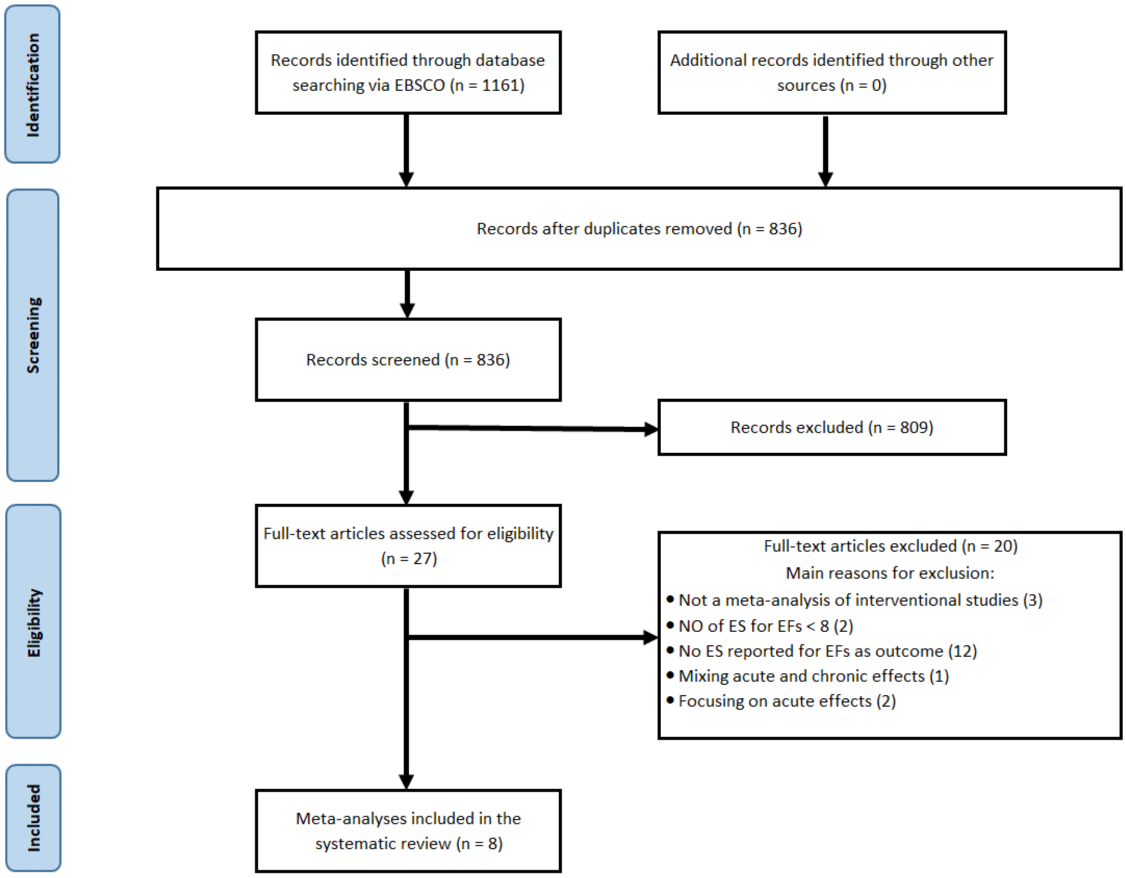


**Figure S5:** Flow chart of meta-analyses selected for the systematic review on the effect of mindfulness training on executive functions

***S2.7. Eligibility criteria specific to the systematic review of meta-analyses concerning the effect of self-control training on self-control capacity***

Meta-analyses including a majority of interventions that did not use self-control tasks were not selected. The five non-selected meta-analyses considered RCT using mindfulness exercises (Leyland et al., 2019) or behavioral change techniques such as self-monitoring, self-reinforcement and/or goal setting (Febbraro & Clum, 1998; Walters, 2000; Reid, Trout, & Schartz, 2005; Leyland, Rowse, & Emerson, 2018). Figure S6 describes the selection process of the systematic review of meta-analyses on the effect self-control training on self-control capacity.

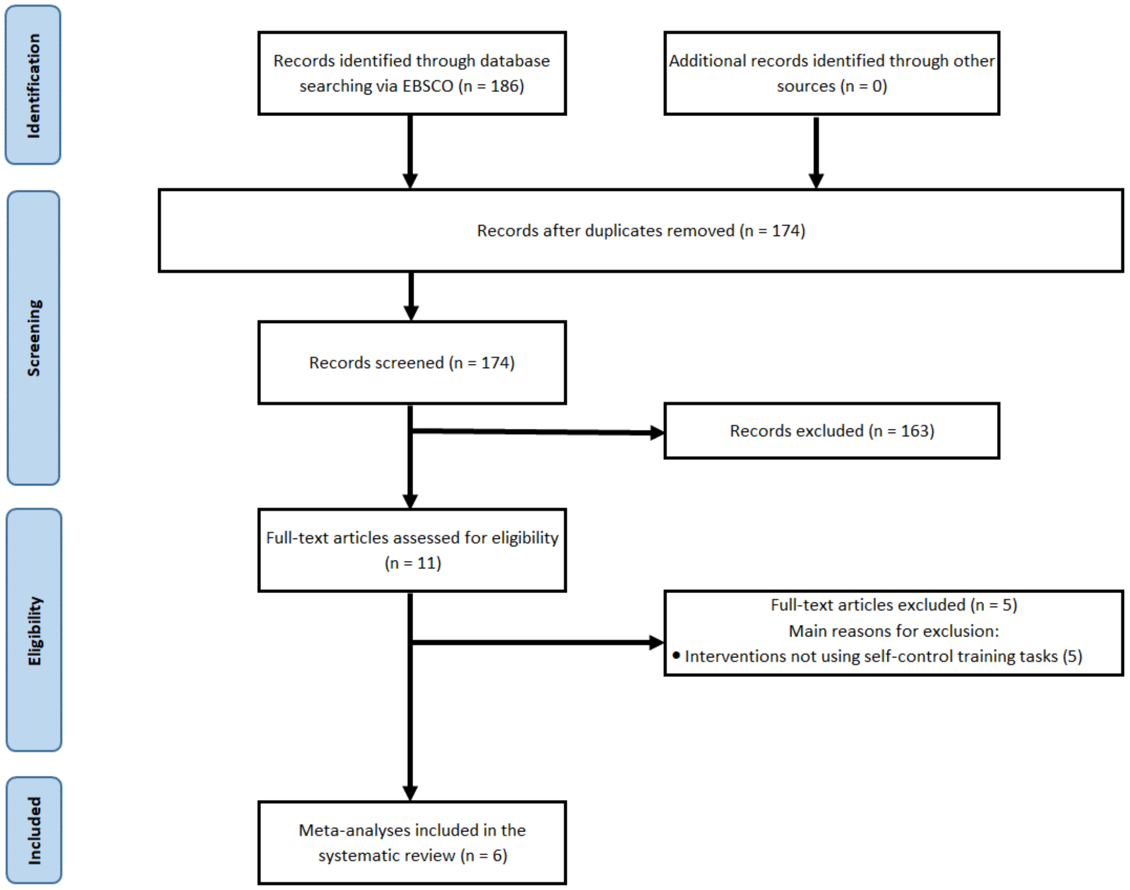


**Figure S6:** Flow chart of meta-analyses selected for the systematic review on the effect of self-control training on self-control capacity

**S3. Data collection and extraction**

Two reviewers were involved in the meta-analysis selection process. The first reviewer (MA) screened eligibility criteria and selected studies for inclusion based on the title and abstract of the article. The second reviewer (NA) checked the inclusion proposed by the first reviewer based on the information. If a disagreement occurred between the two reviewers, a third reviewer (RB) had to examine the eligibility criteria of the concerned study based on the complete article. Then, a debate was organized between the three reviewers to find an agreement. All the references selected from the search process were saved on Zotero. A directory was dedicated to the totality of references obtained through the search process. Another directory was dedicated to the references selected by MA and checked by NA. Figures S1 to S8 describe the selection process of the eight systematic reviews of meta-analyses on the effect of different types of training program on executive functions and self-control.

The following data were extracted from selected studies: name of the first author, year of publication, type of interventions, number of selected studies in the meta-analysis, number of effect sizes included to compute the mean effect size for executive functions, self-control, or a component of executive functions (e.g., inhibitory control, cognitive flexibility), range of duration of the interventions, mean duration of the interventions, population concerned by the meta-analysis, range of age in the targeted population and/or mean age of this population sample, mean effect size for executive functions or for a component of executive functions or self-control, risks of bias.

***S3.1. List and characteristics of included meta-analyses focusing on the effect of interventions combining exercise and process-based cognitive training on executive functions***

| Reference | Interventions | NO studies (A/B) | Population | Duration of interventions | Results |
|---|---|---|---|---|---|
| Zhu et al. (2016) | Combined physical and cognitive training | 12 / 20 | Healthy older adults (Mean age = 71.5 years) | 6-392 hours<br>M = 75.5 hours<br>7-96 weeks<br>M = 18.5 weeks | SMD = 0.13* |
| Guo et al. (2020) | Combined physical and cognitive training | 21 / 21 | Older adults with or without MCI (Mean age = 71.8 years) | 6-160 hours<br>M = 42.5 hours<br>6-24 weeks<br>M = 13.4 weeks | SMD = 0.27* |

Notes: The third column expresses the ratio A / B. The denominator B designates the total number of studies included in the meta-analysis whereas the numerator A designates the number of intervention studies including at least one measurement of executive functions that was used to compute the effect size concerning executive functions. MCI = Mild Cognitive Impairment; NO studies = Number of studies; SMD: Standardized mean difference.

***S3.2. List and characteristics of included meta-analyses focusing on the effect of video game training on executive functions***

| **Reference** | **Interventions** | **NO studies (A/B)** | **Population** | **Duration of interventions** | **Results** |
|---|---|---|---|---|---|
| Stanmore et al. (2017) | Active video game training | 13 / 17 | Adolescents, middle-aged adults and older adults<br>Mean age = 67.2 years | 4-24 weeks<br>M = 10.1 weeks | g = 0.256* |
| Mura et al. (2018) | Active video game training | 8 / 13 | Children, middle-aged adults and older adults<br>Mean age = 52.5 years | 6-48 hours<br>M = 19.7 hours<br>2-24 weeks<br>M = 9.8 weeks | SMD = 0.53* |
| Mansor et al. (2020) | Video game training | ATT: 8 / 27<br>REAS: 10 / 27<br>UWM: 19 / 27<br>IC: 15 / 27<br>CF: 15 / 27 | Older adults<br>Mean age = 71.6 years | 3-60 hours<br>M = 20.8 hours<br>3-16 weeks<br>M = 8.7 weeks | ATT: g = 0.08 ns<br>REA: g = 0.17 ns<br>UWM: g = 0.37*<br>IC: g = 0.28*<br>Adj. IC: g = 0.10 ns<br>CF: g = 0.14 ns |

Notes: The third column expresses the ratio A / B. The denominator B designates the total number of studies included in the meta-analysis whereas the numerator A designates the number of intervention studies including at least one measurement of executive functions that was used to compute the effect size concerning executive functions. NO studies = Number of studies; ATT = Attention; REA = Reasoning; UWM = Updating of working memory; IC = Inhibitory control; Adj. IC = Effect size adjusted for publication bias related to inhibitory control; CF = Cognitive flexibility; SMD: Standardized mean difference.

***S3.3. List and characteristics of included meta-analyses focusing on the effect of mindfulness training on executive functions***

| Reference | Interventions | NO studies (A/B) | Population | Duration of interventions | Results |
|---|---|---|---|---|---|
| Chan et al. (2019) | Meditation-based training | 8 / 41 | Older adults<br>Mean age = 64.6 years | 3-28 hours<br>M = 16.8 hours<br>2-12 weeks<br>M = 7.6 weeks | SMD = 0.29 ns |
| Dunning et al. (2019) | Mindfulness-based training | 14 / 33 | Children and adolescents<br>Mean age = 12.0 years | 1.5-15 hors<br>M = 8.2 hours<br>4-24 weeks<br>M = 9.4 weeks | d = 0.30* |
| Casedas et al. (2020) | Mindfulness-based training | 13 / 13 | Young, middle-aged and older adults<br>Mean age = 45.5 years | 1-636 hours<br>M = 58.6 hours<br>1-12 weeks<br>M = 5.6 weeks | g = 0.34* |
| Poissant et al. (2020) | Mindfulness-based training | 9 / 14 | Young and middle-aged adults with ADHD<br>Mean age = 33.7 years | 12-72 hours<br>M = 30.9 hours<br>1-13 weeks<br>M= 7.2 weeks | g = 0.395* |
| Im et al. (2021) | Mindfulness-based training | 13 / 25 | Young and middle-aged adults<br>Mean age = 32.0 years | 3-68.6 hours<br>M = 18.8 hours | SMD = 0.29* |
| Millett et al. (2021) | Group-based meditation training | 29 / 29 | Young, middle-aged and older adults<br>Mean age = 43.6 years | 1-36 hours<br>M = 14.4 hours<br>1-12 weeks<br>M = 6.1 weeks | g = 0.49* |
| Verhaeghen et al. (2021) | Mindfulness-based training | IC: 26 / 40<br>UWM: 12 / 40<br>CF: 7 / 40<br>SA: 11 / 40 | Young, middle-aged and older adults<br>Mean age = 37.0 years | 0.3-48 hours<br>M = 11.7 hours<br>0.4-16 weeks<br>M = 5.8 weeks | IC: g = 0.32*<br>UWM: g = 0.27*<br>CF: g = 0.33*<br>SA: g = 0.33* |

| | | | | | |
|---|---|---|---|---|---|
| Yakobi et al. (2021) | Mindfulness-based training | 19 / 27 | Young and middle-aged adults<br>Mean age = 26.7 years | 0.5-83 hours<br>M = 18.4 hours<br>1-12 weeks<br>M = 4.5 weeks | g = 0.177* |

Notes: The third column expresses the ratio A / B. The denominator B designates the total number of studies included in the meta-analysis whereas the numerator A designates the number of intervention studies including at least one measurement of executive functions that was used to compute the effect size concerning executive functions. NO studies = Number of studies; SMD: Standardized mean difference.

***S3.4. List and characteristics of included meta-analyses focusing on the effect of self-control training on self-control capacity***

| **Reference** | **Interventions** | **NO studies (A/B)** | **Population** | **Duration of interventions** | **Results** |
|---|---|---|---|---|---|
| Hagger et al. (2010) | Self-control training | 6 / 6 | Young and middle-aged adults | 2-16 weeks<br>M = 5.25 weeks | d = 1.07* |
| Inzlicht & Berkman (2015) | Self-control training | 13 / 13 | Young and middle-aged adults | 1-16 weeks<br>M = 3.92 weeks | d = 0.17 ns |
| Piquero et al. (2016) | Self-control training | 41 / 41 | Children | 6-13 weeks<br>M = 7.09 weeks | g = 0.316* |
| Friese et al. (2017) | Self-control training | 33 / 33 | Young and middle-aged adults | 1-6 weeks<br>M = 2.29 weeks | g = 0.30* |
| Beames et al. (2018) | Self-control training | 27 / 27 | Young and middle-aged adults | 1-16 weeks<br>M = 2.36 weeks | g = 0.36* |
| Pandey et al. (2018) | Self-control training | 19 / 19 | Children | 1-156 weeks<br>M = 30 weeks | d = 0.42* |

Notes: The third column expresses the ratio A / B. The denominator B designates the total number of studies included in the meta-analysis whereas the numerator A designates the number of intervention studies including at least one measurement of executive functions that was used to compute the effect size concerning executive functions. NO studies = Number of studies.

## S4. Percentage of interventional studies shared by meta-analysis datasets

### *S4.1. Meta-analyses on the effect of process-based cognitive training on executive functions*

The 16 meta-analyses listed in table 1 totalize 381 different interventional studies. Among these 381 interventional studies, 75,3% have been selected in only one meta-analysis, 14,2% in 2 meta-analyses, 7,3% in 3 meta-analyses and 3,1% in 4 or 5 meta-analyses.

### *S4.2. Meta-analyses on the effect of exercise training on executive functions*

The 28 meta-analyses listed in table 2 totalize 337 different interventional studies. Among these 337 interventional studies, 55.78% have been selected in only one meta-analysis, 19.36% in 2 meta-analyses, 15.03% in 3 or 4 meta-analyses, 8.38% in 5 or 6 meta-analyses and 1.45% in 7 or 8 meta-analyses.

### *S4.3. Meta-analyses on the effect of interventions combining physical and process-based cognitive training on executive functions*

The two meta-analyses listed in table S3.1 totalize 26 different interventional studies. Among these 26 different interventional studies, 38.46% were duplicated in both meta-analyses.

### *S4.4. Meta-analyses on the effect of video game training on executive functions*

The three meta-analyses listed in table S3.2 totalize 40 different interventional studies. Among these 40 different interventional studies, 17.5% were duplicated among the three meta-analyses. Three interventional studies using exergames were also included in the two meta-analyses focusing on interventions combining physical and process-based cognitive training.

### *S4.5. Meta-analyses on the effect of mindfulness training on executive functions*

The eight meta-analyses listed in table S3.3 totalize 95 different interventional studies. Among these 95 different interventional studies, 68.42% have been selected in only one meta-analysis, 14.74% have been duplicated in two meta-analyses, 10.53% in three meta-analyses and 6.32% in four or five meta-analyses.

### *S4.6. Meta-analyses on the effect of self-control training on self-control capacity*

The percentage of duplicated interventional studies examined by the different selected meta-analyses is reported in the manuscript.

**S5. Quality assessment**

The risk of bias assessment was performed using the AMSTAR2 checklist (Shea et al., 2017). The AMSTAR2 checklist is a measurement tool designed to assess the quality of the execution and reporting of systematic reviews and meta-analyses. The checklist includes 16 criteria listed below. Each criterion is referred to a relevant methodological aspect of the study, thus the quality score for each study could range from 0 to 16.

Two reviewers (MA and NA) read each meta-analysis report and assessed the risk of bias according to the AMSTAR2 checklist. When a disagreement was observed between the two reviewers, a third reviewer had to assess the quality of the study and a debate including the three reviewers followed. The debate systematically concluded by an agreement between the three reviewers. The 16 items of the AMSTAR-2 checklist are listed hereafter. Items 2, 4, 7, 9, 11, 13 and 15 concern critical domains. The quality of the meta-analysis is considered as high (HQ) if there is no more than one non-critical weakness, moderate if there is more than one non-critical weakness (MQ), low if there is only one critical flaw with or without non-critical weaknesses (LQ) and critically low if there is more than one critical flaw with or without non-critical weaknesses (CLQ).

1. Did the research questions and inclusion criteria for the review include the components of PICO?
2. Did the report of the review contain an explicit statement that the review methods were established prior to the conduct of the review and did the report justify any significant deviations from the protocol?
3. Did the review authors explain their selection of the study designs for inclusion in the review?
4. Did the review authors use a comprehensive literature search strategy?
5. Did the review authors perform study selection in duplicate?
6. Did the review authors perform data extraction in duplicate?
7. Did the review authors provide a list of excluded studies and justify the exclusions?
8. Did the review authors describe the included studies in adequate detail?
9. Did the review authors use a satisfactory technique for assessing the risk of bias in individual studies that were included in the review?
10. Did the review authors report on the sources of funding for the studies included in the review?

11. If meta-analysis was performed did the review authors use appropriate methods for statistical combination of results?

12. If meta-analysis was performed, did the review authors assess the potential impact of risk of bias in individual studies on the results of the meta-analysis or other evidence synthesis?

13. Did the review authors account for risk of bias in individual studies when interpreting/ discussing the results of the review?

14. Did the review authors provide a satisfactory explanation for, and discussion of, any heterogeneity observed in the results of the review?

15. If they performed quantitative synthesis did the review authors carry out an adequate investigation of publication bias (small study bias) and discuss its likely impact on the results of the review?

16. Did the review authors report any potential sources of conflict of interest, including any funding they received for conducting the review?

### *S5.1. Risk of bias for meta-analyses on the effect of process-based cognitive training on executive functions*

The average score on the AMSTAR-2 scale was 10.94 / 16 (SD = 1.57). There was no meta-analysis of high quality, but only one meta-analysis of moderate quality (i.e., presenting no critical risk of bias; Basak et al., 2020). All the other meta-analyses were of critically low quality (i.e., presenting two or more critical risk of bias). Among these 15 critically low quality meta-analyses, 75% did not mention an explicit statement that the review method was established before the beginning of the search process (criterion 2), 87.5% did not provide the list of excluded studies (criterion 7), 25% did not use a satisfactory technique for assessing the risk of bias (criterion 9), 68.75% did not account for risk of bias in individual studies when discussing the results of the meta-analysis (criterion 13), and 12.5% did not carry out an adequate investigation of publication bias and discuss it likely impact on the results of the review (criterion 15). Table S1 summarizes the risk of bias for the 16 selected meta-analyses examining process-based cognitive training effects.

| Reference | 1 | 2 | 3 | 4 | 5 | 6 | 7 | 8 | 9 | 10 | 11 | 12 | 13 | 14 | 15 | 16 | Total | Risk of bias |
|---|---|---|---|---|---|---|---|---|---|---|---|---|---|---|---|---|---|---|
| Karch et al. (2013) | 1 | 0 | 1 | 1 | 1 | 1 | 0 | 1 | 1 | 0 | 1 | 0 | 0 | 1 | 0 | 1 | 10 | CLQ |
| Rapport et al. (2013) | 1 | 0 | 1 | 1 | 1 | 1 | 0 | 1 | 0 | 0 | 1 | 0 | 0 | 1 | 1 | 0 | 9 | CLQ |
| Lampit et al. (2014) | 1 | 0 | 1 | 1 | 1 | 1 | 0 | 1 | 1 | 0 | 1 | 1 | 0 | 1 | 1 | 1 | 12 | CLQ |
| Cortese et al. (2015) | 1 | 1 | 1 | 1 | 1 | 1 | 0 | 1 | 1 | 0 | 1 | 0 | 0 | 1 | 1 | 1 | 12 | CLQ |
| Lawrence et al. (2017) | 1 | 0 | 1 | 1 | 1 | 0 | 0 | 1 | 1 | 0 | 1 | 0 | 0 | 1 | 1 | 1 | 10 | CLQ |
| Sherman et al. (2017) | 1 | 0 | 1 | 1 | 1 | 0 | 0 | 1 | 1 | 0 | 1 | 0 | 0 | 1 | 1 | 1 | 10 | CLQ |
| Soveri et al. (2017) | 1 | 0 | 1 | 1 | 0 | 1 | 1 | 1 | 0 | 0 | 1 | 0 | 0 | 1 | 1 | 0 | 9 | CLQ |
| Webb et al. (2018) | 1 | 0 | 1 | 1 | 1 | 1 | 0 | 1 | 1 | 0 | 1 | 0 | 0 | 1 | 0 | 1 | 10 | CLQ |
| Lampit et al. (2019) | 1 | 1 | 1 | 1 | 1 | 1 | 0 | 1 | 1 | 0 | 1 | 1 | 0 | 1 | 1 | 1 | 13 | CLQ |
| Nguyen et al. (2019) | 1 | 0 | 1 | 1 | 1 | 1 | 0 | 1 | 1 | 0 | 1 | 1 | 1 | 1 | 1 | 1 | 13 | CLQ |
| Takacs & Kassai (2019) | 1 | 0 | 1 | 1 | 1 | 1 | 0 | 1 | 0 | 0 | 1 | 0 | 0 | 0 | 1 | 1 | 9 | CLQ |
| Zhang et al. (2019) | 1 | 0 | 1 | 1 | 0 | 0 | 0 | 1 | 1 | 0 | 1 | 0 | 1 | 1 | 1 | 1 | 10 | CLQ |
| Basak et al. (2020) | 1 | 1 | 1 | 1 | 1 | 0 | 1 | 1 | 1 | 0 | 1 | 1 | 1 | 1 | 1 | 1 | 14 | MQ |
| Pauli-Pott et al. (2020) | 1 | 0 | 1 | 1 | 0 | 1 | 0 | 1 | 1 | 0 | 1 | 0 | 1 | 1 | 1 | 1 | 11 | CLQ |
| Scionti et al. (2020) | 1 | 1 | 1 | 1 | 1 | 1 | 0 | 1 | 0 | 0 | 1 | 0 | 0 | 1 | 1 | 1 | 11 | CLQ |
| Nguyen et al. (2021) | 1 | 0 | 1 | 1 | 1 | 0 | 0 | 1 | 1 | 1 | 1 | 0 | 1 | 1 | 1 | 1 | 12 | CLQ |

**Note:** 1: Yes; 0: No; CLQ: Critically Low-quality review; LQ: Low quality review; MQ: Moderate quality review; HQ: High quality review. Critical items are circled.

**Table S1:** Risk of bias according to the AMSTAR-2 scale bias for meta-analyses on the effect of process-based cognitive training on executive functions

***S5.2. Risk of bias for meta-analyses on the effect of exercise training on executive functions***

The average score on the AMSTAR-2 scale was 10.39 / 16 (SD = 2.04). There was no meta-analysis of high or moderate quality (i.e., presenting no critical risk of bias). Only four meta-analyses were of low quality (Karr et al. 2014; Alvarez-Bueno et al., 2017; Biazus-Sehn et al., 2020; Ludyga et al., 2020). The only critical risk of bias they presented was due to the fact that they did not provide the list of excluded studies (criterion 7). All the other meta-analyses were

of critically low quality (i.e., presenting two or more critical risk of bias). Among these 24 critically low quality meta-analyses, 83.3% did not mention an explicit statement that the review method was established before the beginning of the search process (criterion 2), 91.7% did not provide the list of excluded studies (criterion 7), 0.08% (2 meta-analyses) did not use a satisfactory technique for assessing the risk of bias (criterion 9), 75.0% did not account for risk of bias in individual studies when discussing the results of the meta-analysis (criterion 13), and 41.7% did not carry out an adequate investigation of publication bias and discuss it likely impact on the results of the review (criterion 15). Table S2 summarizes the risk of bias for the 27 selected meta-analyses.

| **Reference** | **1** | **2** | **3** | **4** | **5** | **6** | **7** | **8** | **9** | **10** | **11** | **12** | **13** | **14** | **15** | **16** | **Total** | **Risk of bias** |
|---|---|---|---|---|---|---|---|---|---|---|---|---|---|---|---|---|---|---|
| Colcombe & Kramer (2003) | 1 | 0 | 0 | 1 | 0 | 0 | 0 | 0 | 0 | 0 | 1 | 0 | 0 | 0 | 0 | 1 | 4 | CLQ |
| Smith et al. (2010) | 1 | 0 | 1 | 1 | 0 | 1 | 1 | 1 | 1 | 0 | 1 | 0 | 1 | 1 | 0 | 1 | 11 | CLQ |
| Hindin & Zelinski (2011) | 1 | 0 | 1 | 1 | 0 | 0 | 0 | 0 | 1 | 0 | 1 | 0 | 1 | 1 | 0 | 1 | 8 | CLQ |
| Karr et al. (2014) | 1 | 1 | 1 | 1 | 1 | 0 | 1 | 1 | 1 | 0 | 1 | 1 | 1 | 1 | 0 | 1 | 13 | LQ |
| Jackson et al. (2016) | 1 | 1 | 1 | 1 | 1 | 0 | 0 | 1 | 1 | 0 | 1 | 1 | 1 | 1 | 0 | 1 | 12 | CLQ |
| Alvarez-Bueno et al. (2017) | 1 | 1 | 1 | 1 | 1 | 1 | 0 | 1 | 1 | 0 | 1 | 1 | 1 | 1 | 1 | 1 | 14 | LQ |
| Barha et al. (2017) | 1 | 0 | 1 | 1 | 1 | 0 | 0 | 1 | 1 | 0 | 1 | 0 | 0 | 1 | 0 | 1 | 9 | CLQ |
| de Greeff et al (2018) | 1 | 0 | 1 | 1 | 1 | 0 | 1 | 1 | 1 | 0 | 1 | 0 | 0 | 0 | 0 | 1 | 9 | CLQ |
| Northey et al. (2018) | 1 | 0 | 1 | 1 | 1 | 1 | 0 | 1 | 1 | 0 | 1 | 0 | 0 | 0 | 1 | 1 | 10 | CLQ |
| Zhang et al. (2018) | 1 | 0 | 1 | 1 | 1 | 1 | 0 | 1 | 1 | 0 | 1 | 0 | 0 | 1 | 0 | 1 | 10 | CLQ |
| Landrigan et al. (2019) | 1 | 0 | 1 | 1 | 1 | 0 | 0 | 1 | 1 | 0 | 1 | 0 | 0 | 1 | 1 | 0 | 9 | CLQ |
| Falck et al. (2019) | 1 | 0 | 1 | 1 | 1 | 1 | 0 | 1 | 1 | 0 | 1 | 0 | 0 | 1 | 1 | 1 | 11 | CLQ |
| Sanders et al. (2019) | 1 | 1 | 1 | 1 | 1 | 0 | 0 | 1 | 1 | 0 | 1 | 1 | 0 | 1 | 1 | 1 | 12 | CLQ |
| Takacs & Kassai (2019) | 1 | 0 | 1 | 1 | 1 | 1 | 0 | 1 | 0 | 0 | 1 | 0 | 0 | 0 | 1 | 1 | 9 | CLQ |
| Wu et al. (2019) | 1 | 0 | 1 | 1 | 0 | 1 | 0 | 1 | 1 | 0 | 1 | 0 | 1 | 1 | 1 | 1 | 11 | CLQ |
| Xue et al. (2019) | 1 | 0 | 1 | 1 | 1 | 0 | 0 | 1 | 1 | 0 | 1 | 0 | 0 | 1 | 1 | 1 | 10 | CLQ |
| Zou et al. (2019) | 0 | 0 | 1 | 1 | 1 | 1 | 0 | 1 | 1 | 0 | 1 | 1 | 0 | 0 | 1 | 1 | 10 | CLQ |

| | | | | | | | | | | | | | | | | | | |
|---|---|---|---|---|---|---|---|---|---|---|---|---|---|---|---|---|---|---|
| Biazus-Sehn et al. (2020) | 1 | 1 | 1 | 1 | 1 | 1 | 0 | 1 | 1 | 0 | 1 | 1 | 1 | 1 | 1 | 1 | 14 | LQ |
| Cai et al. (2020) | 0 | 0 | 1 | 1 | 1 | 1 | 0 | 1 | 1 | 0 | 1 | 0 | 0 | 0 | 1 | 1 | 9 | CLQ |
| Chen et al. (2020) | 1 | 0 | 1 | 1 | 1 | 1 | 0 | 1 | 1 | 0 | 1 | 0 | 0 | 0 | 1 | 1 | 10 | CLQ |
| Liu et al. (2020) | 1 | 0 | 1 | 1 | 1 | 1 | 0 | 1 | 1 | 0 | 1 | 0 | 0 | 1 | 0 | 1 | 10 | CLQ |
| Ludyga et al. (2020) | 1 | 1 | 1 | 1 | 1 | 1 | 0 | 1 | 1 | 0 | 1 | 1 | 1 | 1 | 1 | 1 | 14 | LQ |
| Zhu et al. (2020) | 1 | 0 | 1 | 1 | 1 | 0 | 0 | 1 | 1 | 0 | 1 | 0 | 1 | 1 | 1 | 1 | 11 | CLQ |
| Dauwan et al. (2021) | 1 | 0 | 1 | 1 | 0 | 0 | 0 | 1 | 1 | 0 | 1 | 1 | 1 | 1 | 1 | 1 | 11 | CLQ |
| Huang et al. (2021) | 1 | 1 | 1 | 1 | 1 | 1 | 0 | 1 | 1 | 0 | 1 | 0 | 0 | 1 | 0 | 1 | 11 | CLQ |
| Ren et al. (2021) | 1 | 0 | 1 | 1 | 1 | 1 | 0 | 1 | 1 | 0 | 1 | 0 | 0 | 0 | 1 | 1 | 10 | CLQ |
| Welsch et al. (2021) | 1 | 1 | 1 | 1 | 0 | 0 | 0 | 1 | 1 | 0 | 1 | 0 | 0 | 1 | 0 | 1 | 9 | CLQ |
| Xiong et al. (2021) | 1 | 0 | 1 | 1 | 1 | 1 | 0 | 1 | 1 | 0 | 1 | 0 | 0 | 0 | 1 | 1 | 10 | CLQ |

**Note:** 1: Yes; 0: No; CLQ: Critically Low-quality review; LQ: Low quality review; MQ: Moderate quality review; HQ: High quality review. Critical items are circled.

**Table S2:** Risk of bias according to the AMSTAR-2 scale bias for meta-analyses on the effect of exercise training on executive functions

### *S5.3. Risk of bias for meta-analyses on the effect of miscellaneous interventions on executive functions*

Given the low number of selected meta-analyses for the effects of combined physical and process-based cognitive training (2); video game training (3), and mindfulness training (8), we decided to gather together 13 meta-analyses in the same table. The three meta-analyses examining the effect of video game training on executive functions are all of critically low quality (M = 9.33 / 16). Among the two meta-analyses examining the effect of combined physical and process-based cognitive training on executive functions, one is of low quality and the other of critically low quality. Finally, among the eight meta-analyses examining the effect of mindfulness training on executive functions, one is of moderate quality, two of low quality and five of critically low quality (M = 12 / 16). Among the thirteen meta-analyses, 69.23% did not pre-register their review methods prior to the conduct of the review (critical criterion 2), 92.31% did

not provide a list of excluded studies and justify their exclusions (critical criterion 7) and 46.15% did not account for risk of bias in individual studies when discussing the results of the review (critical criterion 13). Table S3 summarizes the risk of bias for the 13 selected meta-analyses.

| **Reference** | **1** | **2** | **3** | **4** | **5** | **6** | **7** | **8** | **9** | **10** | **11** | **12** | **13** | **14** | **15** | **16** | **Total** | **Risk of bias** |
|---|---|---|---|---|---|---|---|---|---|---|---|---|---|---|---|---|---|---|
| Meta-analyses on the effect of combined physical and process-based cognitive training on executive functions | | | | | | | | | | | | | | | | | | |
| Zhu et al. (2016) | 1 | 1 | 1 | 1 | 1 | 1 | 0 | 1 | 1 | 0 | 1 | 1 | 1 | 1 | 1 | 1 | 14 | LQ |
| Guo et al. (2020) | 1 | 0 | 1 | 1 | 0 | 0 | 0 | 1 | 1 | 0 | 1 | 1 | 0 | 1 | 1 | 1 | 10 | CLQ |
| Meta-analyses on the effect of video game training on executive functions | | | | | | | | | | | | | | | | | | |
| Stanmore et al. (2017) | 1 | 0 | 1 | 1 | 0 | 1 | 0 | 1 | 1 | 0 | 1 | 0 | 0 | 1 | 1 | 1 | 10 | CLQ |
| Mura et al. (2018) | 1 | 0 | 1 | 1 | 1 | 0 | 0 | 1 | 1 | 0 | 1 | 0 | 0 | 0 | 1 | 1 | 9 | CLQ |
| Mansor et al. (2020) | 1 | 0 | 1 | 1 | 0 | 0 | 0 | 1 | 1 | 0 | 1 | 0 | 0 | 1 | 1 | 1 | 9 | CLQ |
| Meta-analyses on the effect of mindfulness training on executive functions | | | | | | | | | | | | | | | | | | |
| Chan et al. (2019) | 1 | 0 | 1 | 1 | 1 | 1 | 0 | 1 | 1 | 0 | 1 | 1 | 1 | 1 | 1 | 1 | 13 | CLQ |
| Dunning et al. (2019) | 1 | 1 | 1 | 1 | 1 | 0 | 1 | 1 | 1 | 0 | 1 | 1 | 1 | 1 | 1 | 1 | 14 | MQ |
| Casedas et al. (2020) | 1 | 0 | 1 | 1 | 1 | 1 | 0 | 1 | 1 | 0 | 1 | 0 | 1 | 1 | 1 | 1 | 12 | CLQ |
| Poissant et al. (2020) | 1 | 0 | 1 | 1 | 0 | 0 | 0 | 0 | 1 | 0 | 1 | 1 | 1 | 1 | 1 | 1 | 10 | CLQ |
| Im et al. (2021) | 1 | 0 | 1 | 1 | 1 | 1 | 0 | 1 | 1 | 0 | 1 | 0 | 0 | 1 | 1 | 1 | 11 | CLQ |
| Millett et al. (2021) | 1 | 1 | 1 | 1 | 1 | 1 | 0 | 1 | 1 | 0 | 1 | 1 | 1 | 1 | 1 | 1 | 14 | LQ |
| Verhaeghen et al. (2021) | 1 | 0 | 1 | 1 | 0 | 0 | 0 | 1 | 1 | 0 | 1 | 0 | 0 | 1 | 1 | 1 | 9 | CLQ |
| Yakobi et al. (2021) | 1 | 1 | 1 | 1 | 0 | 1 | 0 | 1 | 1 | 0 | 1 | 1 | 1 | 1 | 1 | 1 | 13 | LQ |

**Note:** 1: Yes; 0: No; CLQ: Critically Low-quality review; LQ: Low quality review; MQ: Moderate quality review; HQ: High quality review. Critical items are circled.

**Table S3:** Risk of bias according to the AMSTAR-2 scale bias for meta-analyses examining the effect of miscellaneous interventions on executive functions

***S5.4. Risk of bias for meta-analyses on the effect of self-control training on executive functions***

The average score on the AMSTAR-2 scale was 7.67 / 16 (SD = 2.50). There was no meta-analysis of high, moderate or low quality (i.e., presenting no or just one critical risk of bias). The six meta-analyses were of critically low quality (i.e., presenting two or more critical risk of bias). Among these 6 critically low quality meta-analyses, 66.7% did not mention an explicit statement that the review method was established before the beginning of the search process (criterion 2), 33.3% did not use a comprehensive literature search strategy (criterion 4), 66.7% did not provide the list of excluded studies and did not use a satisfactory technique for assessing the risk of bias (criteria 7 and 9), all the meta-analyses did not account for risk of bias in individual studies when discussing the results of the meta-analysis (criterion 13), and 16.7% did not carry out an adequate investigation of publication bias and discuss it likely impact on the results of the review (criterion 15). Table S6 summarizes the risk of bias for the 6 selected meta-analyses.

| Reference | 1 | 2 | 3 | 4 | 5 | 6 | 7 | 8 | 9 | 10 | 11 | 12 | 13 | 14 | 15 | 16 | Total | Risk of bias |
|---|---|---|---|---|---|---|---|---|---|---|---|---|---|---|---|---|---|---|
| Hagger et al. (2010) | 1 | 0 | 1 | 0 | 0 | 0 | 0 | 0 | 0 | 0 | 1 | 0 | 0 | 1 | 0 | 1 | 5 | CLQ |
| Inzlicht & Berkman (2015) | 0 | 0 | 0 | 0 | 0 | 0 | 1 | 1 | 0 | 0 | 1 | 0 | 0 | 0 | 1 | 1 | 5 | CLQ |
| Piquero et al. (2016) | 0 | 0 | 1 | 1 | 0 | 1 | 0 | 1 | 1 | 0 | 1 | 0 | 0 | 0 | 1 | 0 | 7 | CLQ |
| Friese et al. (2017) | 1 | 1 | 1 | 1 | 0 | 0 | 1 | 1 | 0 | 0 | 1 | 0 | 0 | 1 | 1 | 1 | 10 | CLQ |
| Beames et al. (2018) | 1 | 0 | 1 | 1 | 0 | 1 | 0 | 0 | 0 | 0 | 1 | 0 | 0 | 1 | 1 | 1 | 8 | CLQ |
| Pandey et al. (2018) | 1 | 1 | 1 | 1 | 1 | 0 | 0 | 1 | 1 | 0 | 1 | 0 | 0 | 1 | 1 | 1 | 11 | CLQ |

**Note:** 1: Yes; 0: No; CLQ: Critically Low-quality review; LQ: Low quality review; MQ: Moderate quality review; HQ: High quality review. Critical items are circled.

**Table S4:** Risk of bias according to the AMSTAR-2 scale bias for meta-analyses examining the effect of self-control training on self-control capacity
